# BioDynaMo: a modular platform for high-performance agent-based simulation

**DOI:** 10.1093/bioinformatics/btab649

**Published:** 2021-09-16

**Authors:** Lukas Breitwieser, Ahmad Hesam, Jean de Montigny, Vasileios Vavourakis, Alexandros Iosif, Jack Jennings, Marcus Kaiser, Marco Manca, Alberto Di Meglio, Zaid Al-Ars, Fons Rademakers, Onur Mutlu, Roman Bauer

**Affiliations:** CERN openlab, IT Department, CERN, Geneva 1211, Switzerland; Department of Computer Science, ETH Zurich, Zurich 8092, Switzerland; CERN openlab, IT Department, CERN, Geneva 1211, Switzerland; Department of Quantum & Computer Engineering, Delft University of Technology, Delft 2628CD, The Netherlands; CERN openlab, IT Department, CERN, Geneva 1211, Switzerland; Department of Mechanical & Manufacturing Engineering, University of Cyprus, Nicosia 2109, Cyprus; Department of Medical Physics & Biomedical Engineering, University College London, London WC1E 6BT, UK; Department of Mechanical & Manufacturing Engineering, University of Cyprus, Nicosia 2109, Cyprus; School of Computing, Newcastle University, Newcastle upon Tyne NE4 5TG, UK; School of Computing, Newcastle University, Newcastle upon Tyne NE4 5TG, UK; Department of Functional Neurosurgery, Ruijin Hospital, Shanghai Jiao Tong University School of Medicine, Shanghai 200025, China; Precision Imaging Beacon, School of Medicine, University of Nottingham, Nottingham NG7 2UH, UK; SCimPulse Foundation, Geleen 6162 BC, The Netherlands; CERN openlab, IT Department, CERN, Geneva 1211, Switzerland; Department of Quantum & Computer Engineering, Delft University of Technology, Delft 2628CD, The Netherlands; CERN openlab, IT Department, CERN, Geneva 1211, Switzerland; Department of Computer Science, ETH Zurich, Zurich 8092, Switzerland; Department of Information Technology and Electrical Engineering, ETH Zurich, Zurich 8092, Switzerland; Department of Computer Science, University of Surrey, Guildford GU2 7XH, UK

## Abstract

**Motivation:**

Agent-based modeling is an indispensable tool for studying complex biological systems. However, existing simulation platforms do not always take full advantage of modern hardware and often have a field-specific software design.

**Results:**

We present a novel simulation platform called BioDynaMo that alleviates both of these problems. BioDynaMo features a modular and high-performance simulation engine. We demonstrate that BioDynaMo can be used to simulate use cases in: neuroscience, oncology and epidemiology. For each use case, we validate our findings with experimental data or an analytical solution. Our performance results show that BioDynaMo performs up to three orders of magnitude faster than the state-of-the-art baselines. This improvement makes it feasible to simulate each use case with one billion agents on a single server, showcasing the potential BioDynaMo has for computational biology research.

**Availability and implementation:**

BioDynaMo is an open-source project under the Apache 2.0 license and is available at www.biodynamo.org. Instructions to reproduce the results are available in the supplementary information.

**Supplementary information:**

Available at https://doi.org/10.5281/zenodo.5121618.

## 1 Introduction

Agent-based simulation (ABS) is a powerful tool assisting life scientists in better understanding complex biological systems. *In silico* simulation is an inexpensive and efficient way to rapidly test hypotheses about the (patho)physiology of cellular populations, tissues, organs or entire organisms (Ji *et al.*, 2017; Yankeelov *et al.*, 2016).

However, the effectiveness of such computer simulations for scientific research is often limited, in part because of two reasons. First, after the slowing down of Moore’s law (Moore, 1965) and Dennard scaling (Dennard *et al.*, 1974), hardware has become increasingly parallel and heterogeneous. Most ABS platforms do not take full advantage of these hardware enhancements. The resulting limited computational power forces life scientists to compromise either on the resolution of the model or on simulation size (Thorne *et al.*, 2007). Second, existing ABS platforms have often been developed with a specific use case in mind. This makes it challenging to implement the desired model, even if it deviates only slightly from its original purpose.

To help researchers tackle these two major challenges, we propose a novel open-source platform for biology dynamics modeling, BioDynaMo. We alleviate both of these problems by emphasizing performance and modularity. BioDynaMo features a high-performance simulation engine that is fully parallelized to utilize multi-core CPUs and able to offload computation to hardware accelerators (e.g. a GPU). The software comprises a set of fundamental biological functions, and a flexible design that adapts to specific user requirements. Currently, BioDynaMo implements the neurite model and mechanical forces presented in Zubler and Douglas (2009), but these components can easily be extended, modified or replaced. Hence, BioDynaMo is well-suited for simulating a wide range of biological processes in tissue modeling and beyond.

BioDynaMo provides by design five system properties:

- **Agent-based.** The BioDynaMo project is established to support an agent-based modeling approach which allows one to simulate a wide range of developmental biological processes. A characteristic property of agent-based simulations is the absence of a central organizational unit that orchestrates the behavior of all agents. Quite to the contrary, each agent is an autonomous entity that individually determines its actions based on its current state, behavior and the surrounding environment.
- **General purpose.** BioDynaMo is developed to become a general-purpose tool for agent-based simulation. To simulate models from various fields, BioDynaMo’s software design is extensible and modular.
- **Large scale.** Biological systems contain a large number of agents. The cerebral cortex, for example, comprises approximately 16 billion neurons (Azevedo *et al.*, 2009). Biologists should not be limited by the number of agents within a simulation. Consequently, BioDynaMo is designed to take full advantage of modern hardware and use memory efficiently to scale-up simulations.
- **Easily programmable.** The success of an ABS platform depends, among other things, on how quickly a scientist, not necessarily an expert in computer science or high-performance programming, can translate an idea into a simulation. This characteristic can be broken down into four key requirements that BioDynaMo is designed to fullfil: first, BioDynaMo provides a wide range of common functionalities such as visualization, plotting, parameter parsing, backups, etc. Second, BioDynaMo provides simulation primitives that minimize the programming effort necessary to build a use case. Third, as outlined in item ‘General purpose’, BioDynaMo has a modular and extensible design. Fourth, BioDynaMo provides a coherent API and hides implementation details that are irrelevant for a computational model (e.g. details such as parallelization strategy, synchronization, load balancing or hardware optimizations).
- **Quality assured.** BioDynaMo establishes a rigorous, test-driven development process to foster correctness, maintainability of the codebase and reproducibility of results.

The main contribution of this article is an open-source, high-performance and modular simulation platform for agent-based simulations. We provide the following evidence to support this claim: (i) we detail the user-facing features of BioDynaMo that enable users to build a simulation based on predefined building blocks and to define a model tailored to their needs. (ii) We present three basic use cases in the field of neuroscience, oncology and epidemiology to demonstrate BioDynaMo’s capabilities and modularity. (iii) We show that BioDynaMo can produce biologically meaningful simulation results by validating these use cases against experimental data, or an analytical solution. (iv) We present performance data on different systems and scale each use case to one billion agents to demonstrate BioDynaMo’s performance.

### 1.1 Prior work

The history of agent-based modeling and simulation significantly precedes the 1990s; however, it was not widely adopted for biological systems until the 2000s. Several ABS platforms have been published demonstrating the importance of agent-based models in computational biology research (Collier and North, 2011; Cytowski and Szymanska, 2014; Emonet *et al.*, 2005; Ghaffarizadeh *et al.*, 2018; Kang *et al.*, 2014; Koene *et al.*, 2009; Lardon *et al.*, 2011; Matyjaszkiewicz *et al.*, 2017; Mirams *et al.*, 2013; Richmond *et al.*, 2010; Rudge *et al.*, 2012; Torben-Nielsen and De Schutter, 2014; Wilensky, 1999; Zubler and Douglas, 2009). In this section, we compare BioDynaMo’s most crucial system properties with prior work.

####  

##### Large-scale model support

The authors of BioCellion (Kang *et al.*, 2014), PhysiCell (Ghaffarizadeh *et al.*, 2018), Timothy (Cytowski and Szymanska, 2014) and Repast HPC (Collier and North, 2011) recognize the necessity for efficient implementations to enable large-scale models. Although these tools can simulate a large number of agents, they do not support neural development. The NeuroMaC neuroscientific simulation platform (Torben-Nielsen and De Schutter, 2014) claims to be scalable, but the authors do not present performance data and present simulations with only 100 neurons. Therefore, BioDynaMo’s ability to simulate large-scale neural development, which we demonstrate in the results section, is, to our knowledge, unrivaled.

##### General-purpose platform

Many ABS platforms focus on a specific application area: bacterial colonies (Emonet *et al.*, 2005; Lardon *et al.*, 2011; Matyjaszkiewicz *et al.*, 2017; Rudge *et al.*, 2012), cell colonies (Cytowski and Szymanska, 2014; Kang *et al.*, 2014; Mirams *et al.*, 2013) and neural development (Koene *et al.*, 2009; Torben-Nielsen and De Schutter, 2014; Zubler and Douglas, 2009). Pronounced specialization of an ABS platform may prevent its capacity to adapt to different use cases or simulation scenarios. In contrast, BioDynaMo can be adapted to many different fields due to its modularity and extensibility.

##### Quality assurance

Automated software testing is the foundation of a modern development workflow. Unfortunately, several simulation tools (Cytowski and Szymanska, 2014; Koene *et al.*, 2009; Lardon *et al.*, 2011; Rudge *et al.*, 2012; Torben-Nielsen and De Schutter, 2014; Zubler and Douglas, 2009) omit these tests. Mirams *et al.* (2013) recognize this shortcoming and describe a rigorous development workflow in their article. BioDynaMo has over 400 automated tests which are continuously executed on all supported operating systems to ensure high code quality. BioDynaMo’s open-source code base, tutorials and documentation not only help users get started, but also enable validation by external examiners.

## 2 Materials and methods

In this section, we present the main simulation concepts of BioDynaMo and describe our approach to achieve modularity, extensibility and high performance.

### 2.1 Simulation concepts

**Fig. 1.**
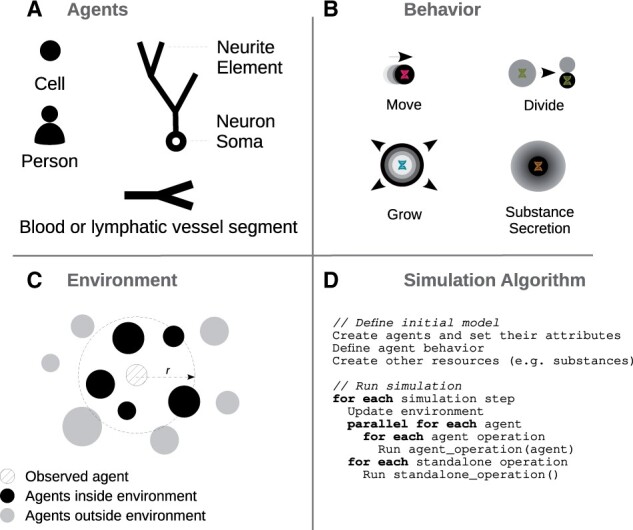
Simulation concepts. Agents (**A**) have their own geometry, behavior (**B**) and environment (**C**). (B) Agent behavior is defined in separate classes, which are inserted into agents. The update of an agent is based on its current state and its surrounding environment. (C) The environment is determined by radius *r* and contains other agents or extracellular substances. The simulation algorithm (**D**) can be divided into two main parts: the definition of the initial model and the execution of the simulation

**Fig. 2.**
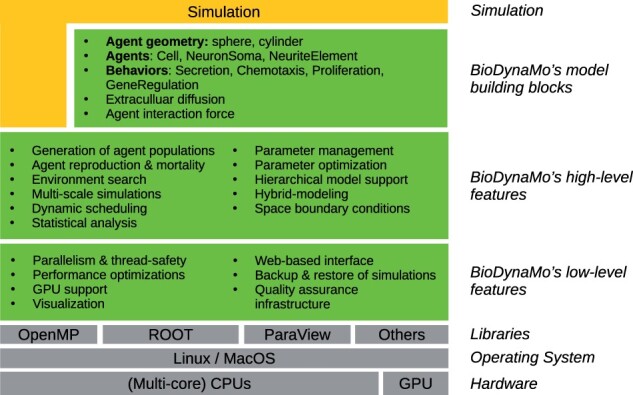
BioDynaMo’s layered architecture. BioDynaMo is predominantly executed on multi-core CPUs, is able to offload computations to the GPU, and supports Linux and MacOS operating systems. BioDynaMo provides a rich set of low- and high-level features commonly required in agent-based models. On top of these generic features, BioDynaMo offers model building blocks to simplify the development of a simulation. Even if BioDynaMo does not provide the required building blocks, users still benefit from all generic agent-based features (illustrated by the vertical extension of the ‘Simulation’ layer)

**Fig. 3.**
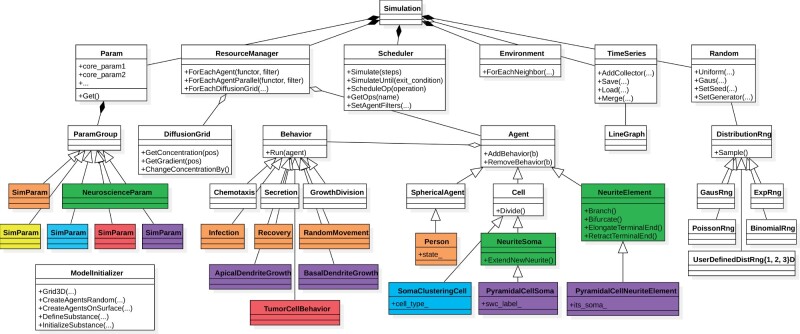
Software design and modularity. Overview of selected classes and functions that are important from the users’ perspective. Classes in white (BioDynaMo core) and green (BioDynaMo’s neuroscience module) are part of the current BioDynaMo installation. The remaining classes illustrate how we extended BioDynaMo to implement the use cases and benchmarks presented in this article (purple: neuroscience use case, red: oncology use case, orange: epidemiology use case, blue: soma clustering benchmark, yellow: cell proliferation benchmark). A complete list of BioDynaMo classes can be found at https://biodynamo.org/api

BioDynaMo is implemented in the C++ programming language and supports simulations that follow an agent-based approach. Figure 1 gives an overview of BioDynaMo’s main concepts, Figure 2 presents the abstraction layers, while Figure 3 illustrates its object-oriented design.

An agent (Figure 1A) has a 3D geometry, behavior and environment. There is a broad spectrum of entities that can be modeled as an agent. In the results section, we show examples where an agent represents a subcellular structure (neuroscience use case), a cell (oncology use case) or a person (epidemiology use case). Figure 1B shows example agent behaviors such as growth factor secretion, chemotaxis or cell division. Behaviors can be activated or inhibited. BioDynaMo achieves this by attaching them to or removing them from the corresponding agent.

The *Environment* is the vicinity that the agent can interact with (Figure 1C). It comprises agents and other resources like chemical substances in the extracellular matrix. Surrounding agents are, for example, needed to calculate mechanical interactions among agent pairs.

Currently, the user defines a simulation programmatically in C++ (see Figure 1D and Supplementary File SF1 Section 1.4). C++ is a great choice in terms of execution speed, efficiency, and interoperability with high-performance computing libraries, but is harder to program in due to its lower level of abstraction (versus higher level languages like Java or Python). There are two main steps involved: initialization and execution. During initialization, the user places agents in space, sets their attributes, and defines their behavior. In the execution step, the simulation engine evaluates the defined model in the simulated physical environment by executing a series of operations. We distinguish between agent operations and standalone operations (Figure 1D). At a high level, an agent operation is a function that: (i) alters the state of an agent and potentially also its environment, (ii) creates a new agent or (iii) removes an agent from the simulation. Examples for agent operations are: execute all behaviors and calculate mechanical forces. The simulation engine executes agent operations for each agent for each time step. Alternatively, standalone operations perform a specific task during one time step and are therefore only invoked once. Examples include the update of substance diffusion and the export of visualization data.

### 2.2 BioDynaMo features

BioDynaMo is a simulation platform that can be used to develop agent-based simulations in various computational biology fields (e.g. neuroscience, oncology, epidemiology, etc.). Although agent-based models in these different fields may intrinsically vary, there is a set of functionalities and definitions that they have in common.

These commonalities can be divided into low- and high-level agent-based features and are an integral part of BioDynaMo. BioDynaMo also provides model building blocks to accelerate the development of agent-based models. The described layered architecture is shown in Figure 2.

#### 2.2.1 Low-level features

The low-level features (Figure 2) form the foundation of BioDynaMo and provide crucial functionality responsible for high-performance execution and ease-of use. These features are mostly hidden from the user and require control only in exceptional situations.

In this section we will provide more details about parallelization and thread safety and refer the reader to Supplementary File SF1 Section 1.1 for more details of the remaining low-level features.

##### Parallelism and thread safety

BioDynaMo exploits the inherent parallelism of agent-based models in which agents update themselves based on their current state, behavior and local environment. BioDynaMo’s implementation uses OpenMP (https://www.openmp.org/) compiler directives to parallelize the loop over all agents (Figure 1D). In addition to parallelizing the execution of agent operations for each agent, standalone operations like updating the diffusion grid and visualization are parallelized separately (Figure 1D).

Synchronization between threads is only needed if agents modify their environment. In this case, two agents (handled by two different threads) might attempt to update the same resource in the local environment. This scenario occurs in the neuroscience use case in which neurite elements modify neighboring segments. Therefore, BioDynaMo provides built-in synchronization mechanisms to ensure that even if two threads try to modify the same agent or resource, data is not corrupted. There are two thread safety mechanisms to protect agents from data corruption: automatic and user-defined. Automatic thread safety uses the environment to prevent two threads from processing agents with overlapping local environments. This mechanism can be enabled with a single parameter, but might be too restrictive for some use cases. User-defined thread safety on the other hand offers more fine-grained control over which agents must not be processed at the same time, but likely requires additional input from the user.

Other resources that are modified by agents (e.g. the DiffusionGrid to simulate extracellular diffusion) need their own protection mechanism. This feature is used in the soma clustering benchmark where cells secrete a substance into the extracellular matrix.

For typical BioDynaMo simulations users do not need to control parallel execution and thread synchronization. This holds particularly true for all use cases and benchmarks presented in this article. Only for more advanced uses, like adding a new environment algorithm or adding a shared resource that is *not* an agent, users have to consider parallel execution.

#### 2.2.2 High-level features

The high-level layer (Figure 2) provides general functionality which is commonly required in agent-based models across many fields.

##### Generation of agent populations

The first step in an agent-based model is to specify the starting condition of the simulation. Therefore, BioDynaMo provides functionality to create agent populations with specific properties. Class ModelInitializer provides several functions to create agents in 3D space and to initialize extracellular substances (Figure 3 and Supplementary File SF1 Section 1.4). Furthermore, to initialize the attributes of an agent population, researchers can use one of the many predefined random number generators that draw samples from a specific distribution (uniform, exponential, gaussian, binomial, etc.) or define their own one. These features are demonstrated in Supplementary Tutorial ST01, ST02 and ST08.

##### Agent reproduction and mortality

The addition and removal of agents during the execution of a simulation is an integral part of agent-based simulations. Therefore, BioDynaMo provides a framework to create new agents during a simulation and initialize their attributes. By default, agents that are created in iteration i will be visible to other agents in iteration i + 1. The removal of agents is handled identically. The handling of when new or removed agents become visible to the simulation is encapsulated in the execution context. Therefore, a user could provide another implementation where agents are visible immediately.

Besides adding and removing agents, a second major part is to provide a generic way to initialize the attributes of an agent. To this end, BioDynaMo simplifies the regulation of behaviors if new agents are created. The user can control whether a behavior will be copied to a new agent or removed from the existing agent, based on the underlying process (e.g. cell division). Similarly, agents have a function Initialize which can be overridden by user-defined agents to initialize additional attributes. These concepts are demonstrated in Supplementary Tutorial ST03–ST05.

##### Environment search

To determine the agents in the local environment (neighbors), BioDynaMo uses an environment search algorithm (Supplementary Tutorial ST06). BioDynaMo’s default environment algorithm is based on a uniform grid implementation. The implementation divides the total simulation space into uniform boxes of the same size and assigns agents to boxes based on the center of mass of the agent. Hence, the agents in the environment can be obtained by iterating over the assigned box and all its surrounding boxes (27 boxes in total). The box size is chosen by the user or determined automatically based on the largest agent in the simulation to ensure all mechanical interactions are taken into account. Alternatively, BioDynaMo provides an octree and kd-tree environment implementation. The interface is kept generic enough to support non-Euclidean space environment definitions.

##### Multi-scale simulations

A biological simulation has to account for dynamic mechanisms that range from milliseconds to weeks (e.g. physical forces, reaction-diffusion processes, gene regulatory dynamics, etc.). BioDynaMo supports processes at different time scales by providing a parameter to specify the time interval between two time steps and an execution frequency for each operation (Supplementary Tutorial ST07). An execution frequency of one means that the corresponding operation is executed every time step. In contrast, a frequency of three would mean that the operation is executed every three time steps. This mechanism allows BioDynaMo to simulate e.g. substance diffusion and neurite growth in the same model.

##### Statistical analysis

Statistical analysis plays a fundamental role in generating new insights from simulation data. BioDynaMo builds upon the rich features of CERN’s primary data analysis framework ROOT (Brun and Rademakers, 1997), which provides an extensive mathematical, histogram, graphing, and fitting library. BioDynaMo complements this functionality by providing an easy mechanism to collect simulation data over time and a simplified API targeted to the agent-based use case. These capabilities are demonstrated in Supplementary Tutorial ST08–ST11.

##### Hierarchical model support

Railsback *et al.* (2006) describe an agent-based model in which large agents have to be executed before smaller agents. BioDynaMo supports these hierarchical models with several functions in the ResourceManager, Scheduler, and Operation class. The described order can be implemented in BioDynaMo by adding three lines of code as demonstrated in Supplementary Tutorial ST12. Additionally, it is possible to execute a different set of operations for large and small agents.

##### Hybrid modeling support

Some models benefit from the combination of multiple simulation methodologies—e.g. the combination of an agent-based and continuum-based model. BioDynaMo’s flexible build system supports hybrid modeling capabilities and was demonstrated by de Montigny *et al.* (2021) to investigate cancer development.

The remaining high-level features are detailed in Supplementary File SF1 Section 1.2.

#### 2.2.3 Model building blocks

Currently, BioDynaMo’s building blocks (Figure 2) belong to the (neural) tissue modeling domain. Similar to the biological model presented in (Zubler and Douglas, 2009), BioDynaMo supports spherical and cylindrical agent geometries, mechanical interactions between agents and diffusion of extracellular substances.

With these features, researchers can simulate cell body dynamics, neural growth and gene regulatory networks. We provide more details about the individual model building blocks in Supplementary File SF1 Section 1.3.

Simulations to study the development of (neural) tissue are only one example of how BioDynaMo could be used in the future. By designing BioDynaMo in a modular and extensible way, we laid the foundation to create new building blocks easily (Figure 3 and Section 3.3).

### 2.3 Use cases

We demonstrate BioDynaMo’s capacity to simulate disparate problems in systems biology with simple yet representative use cases in neuroscience, oncology and epidemiology. Since BioDynaMo does not contain any epidemiological building blocks, this use case indicates how easy it is to implement a model-based solely on features from the high- and low-level layer (Figure 2).

For each use case we present the implemented model, validate the simulation results against verified experimental or analytical data, and report performance data for different problem sizes on multiple hardware configurations. Furthermore, we provide pseudocode for all agent behaviors, a table with model parameters and more detailed performance results in Supplementary File SF1 Section 2.

### 2.4 Performance analysis

We compare BioDynaMo’s performance with two established serial ABS platforms [Cortex3D (Zubler and Douglas, 2009) and NetLogo (Wilensky, 1999)], analyze BioDynaMo’s scalability, and quantify the impact of GPU acceleration. Display updates are turned off on all platforms for these evaluations. Cortex3D has the highest similarity in terms of the underlying biological model out of all the related works presented in Section 1.1. More specifically, BioDynaMo and Cortex3D use the same method to determine mechanical forces between agents and the same model to grow neural morphologies. This makes Cortex3D the best candidate with which to compare BioDynaMo and ensure a fair comparison.

We quantify the performance of BioDynaMo with four simulations: cell growth and division, soma clustering, pyramidal cell growth and the epidemiology use case. We compare the runtime of the first three simulations with Cortex3D and the epidemiology use case with NetLogo 3D. These simulations have different properties and are, therefore, well suited to evaluate BioDynaMo’s simulation engine under a broad set of conditions. Supplementary File SF1 Section 2.2 contains more details about these benchmarks.

####  

##### Statistical method

We perform five measurements for each presented data point in Figure 7 and Table 1. We summarize runtimes using the arithmetic mean and rates such as speedup using the harmonic mean.

**Table 1.** Performance data

Simulation	Agents	Diffusion volumes	Iterations	System	Physical CPUs	Runtime	Memory
Neuroscience use case							
Single (Figure 4A)	1494	250	500	A	1	0.16 s	382 MB
Large-scale (Figure 4C)	9 054 740	65 536	500	A	72	36 s	6.02 GB
Very-large-scale	1 018 644 154	5 606 442	500	B	72	1 h 26 min	436 GB
Oncology use case (Figure 5)							
2000 initial cells	4177	0	312	A	1	1.05 s	382 MB
Large-scale	10 003 925	0	288	A	72	1 min 42 s	7.42 GB
Very-large-scale	986 054 868	0	288	B	72	6 h 21 min	604 GB
Epidemiology use case (Figure 6C)							
Measles	2010	0	1000	A	1	0.53 s	381 MB
Seasonal Influenza	20 200	0	2500	A	1	16.41 s	383 MB
Large-scale (measles)	10 050 000	0	1000	A	72	59.19 s	5.87 GB
Very-large-scale (measles)	1 005 000 000	0	1000	B	72	2 h 0 min	495 GB

*Note*: The values in column ‘Agents’ and ‘Diffusion volumes’ are taken from the end of the simulation. Runtime measures the wall-clock time to simulate the number of iterations. It excludes the time for simulation setup and visualization. The entries in column ‘System’ correspond to Supplementary File SF1 Table 5. Supplementary File SF1 Table 6 contains more detailed performance data.

### 2.5 Reproducibility

We use the latest BioDynaMo version v1.01-55-gd05111e3 for all use cases and benchmarks in the result section. To help other researchers replicate our findings we provide the following Supplementary Information for utmost transparency. First, we publish all source code and data in Supplementary File SF3. The archive contains six shell scripts that execute all simulations, and generate all plots, visualizations and videos shown in this article. Second, we provide a ready-to-use self-contained Docker image to simplify the process of executing our simulations and benchmarks and to guarantee long-term reproducibility (Supplementary File SF4). Third, we add a step-by-step instruction in Supplementary File SF2.

## 3 Results

### 3.1 Neuroscience use case

This example illustrates the use of BioDynaMo to model neurite growth of pyramidal cells using chemical cues. Initially, a pyramidal cell, composed of a 10 μm cell body, three 0.5 μm long basal dendrites and one 0.5 μm long apical dendrite (all of them considered here as agents), is created in 3D space [L37–L51 (Line numbers in Section 3.1 correspond to the code example in Supplementary File SF1 Listing 4.)]. Furthermore, two artificial growth factors were initialized, following a Gaussian distribution along the *z*-axis (L54–L65). The distribution of these growth factors guided dendrite growth and remained unchanged during the simulation.

Dendritic development was dictated by a behavior defining growth direction, speed and branching behavior for apical and basal dendrites (L12–L35). At each step of the simulation, the dendritic growth direction depended on the local chemical growth factor gradient, the dendrite’s previous direction and a randomly chosen direction. In addition, the dendrite’s diameter tapered as it grew (shrinkage), until it reached a specified diameter, preventing it from growing any further. The weight of each element on the direction varied between apical and basal dendrites.

**Fig. 4.**
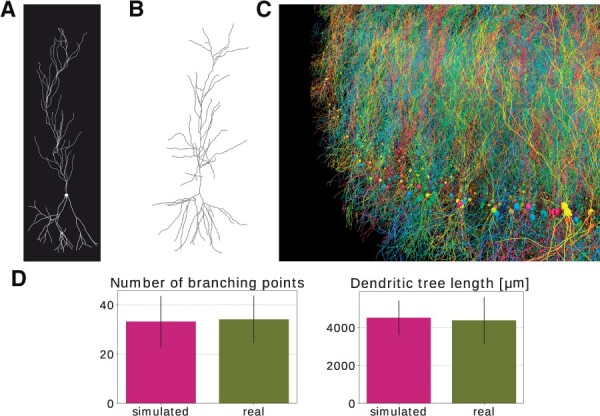
Pyramidal cell simulation. (**A**) Example pyramidal cell simulated with BioDynaMo. (**B**) Real neuron (R67nr67b-CEL1.CNG) taken from Mellström *et al.* (2016) and visualized with https://neuroinformatics.nl/HBP/morphology-viewer/. (**C**) Large-scale simulation. The model started with 5000 initial pyramidal cell bodies and contained 9 million agents after simulating 500 iterations. The simulation execution time was 35 s on a server with 72 CPU cores. (**D**) Morphology comparison between simulated neurons and experimental data from Mellström *et al.* (2016). Error bars represent the standard deviation. (A, C) A video is available in the Supplementary Materials

These simple rules gave rise to a straight long apical dendrite with a simple branching pattern and more dispersed basal dendrites, as shown in Figure 4A, similar to what can be observed in real pyramidal cell morphologies as shown in Figure 4B or Spruston (2008) (Figure 1A CA1). Using our growth model, we were able to generate a large number of various realistic pyramidal cell morphologies. We used a publicly available database of real pyramidal cells coming from Mellström *et al.* (2016) for comparison and parameter tuning. Two measures were used to compare our simulated neurons and the 69 neurons composing the real morphologies database: the average number of branching points, and the average length of dendritic trees. No significant differences were observed between our simulated neurons and the real ones (*P *<* *0.001 using a T-test for two independent samples). These results are shown in Figure 4D. The model specification of the pyramidal cell growth simulation consists of 127 lines of C++ code (Supplementary File SF1 Listing 4).

Figure 4C shows a large-scale simulation incorporating 5000 neurons similar to the one described above and demonstrates the potential of BioDynaMo for developmental, anatomical and connectivity studies in the brain. This simulation contained nine million agents.

### 3.2 Oncology use case

In this section, we present a tumor spheroid simulation to replicate in vitro experiments from Gong *et al.* (2015). Tumor spheroid experiments are typically employed to investigate the pathophysiology of cancer, and are also being used for pre-clinical drug screening (Nunes *et al.*, 2019). Here we considered three in vitro test cases using a breast adenocarcinoma MCF-7 cell line (Gong *et al.*, 2015) with different initial cell populations (2000, 4000 and 8000 MCF-7 cells). Our goal was to simulate the growth of this mono cell culture embedded in a collagenous (extracellular) matrix.

The fundamental cellular mechanisms modeled here include cell growth, cell duplication, cell migration and cell apoptosis. All these processes are implemented in the class TumorCellBehavior. The cell growth rate was derived from the published data (Sutherland *et al.*, 1983), while cell migration (cell movement speed), cell survival and apoptosis were fine-tuned after trial and error testing. Since the in vitro study considered the same agarose gel matrix composition among the experiments, the BioDynaMo model assumes identical parameters for the cell–matrix interactions in the simulations. Considering the homogeneous ECM properties, tumor cell migration was modeled as Brownian motion.

**Fig. 5.**
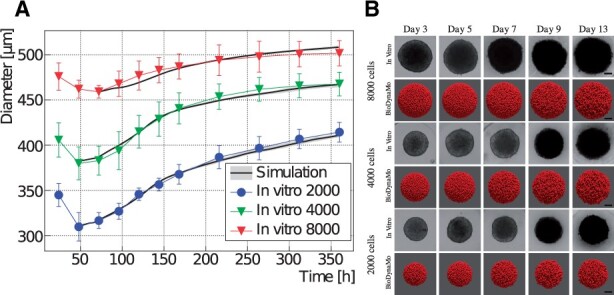
Comparison between in vitro MCF-7 tumor spheroid experiments and our *in silico* simulations using BioDynaMo. (**A**) Human breast adenocarcinoma tumor spheroid (MCF-7 cell line) development during a 15 day period, where different initial cell populations were considered [see Fig. 3 in Gong *et al.* (2015)]. Error bars denote standard deviation to the experimental data. The mean of the *in silico* results is shown as a solid black line with a gray band depicting minimum and maximum observed value. (**B**) A qualitative comparison between the microscopy images and simulation snapshots. Scale bars correspond to 100 μm. A video is available in the Supplementary Materials

The in vitro experiments from Gong *et al.* (2015) and the simulations using BioDynaMo are depicted in Figure 5. Each line plot in Figure 5A compares the mean diameter between the experiments and the simulations over time, which demonstrates the validity and accuracy of BioDynaMo. The diameter of the spheroids in the simulations were deducted from the volume of the convex hull that enclosed all cancer cells. The in vitro experiments used microscopy imaging to measure the spheroid’s diameters (Gong *et al.*, 2015). Figure 5B compares snapshots of the simulated tumor spheroids (bottom row) against microscopy images of in vitro spheroids (top row) at different time points. The spheroid’s morphologies between the in vitro experiments and the BioDynaMo simulations are in excellent agreement.

Model specification required 154 lines of C++ code.

### 3.3 Epidemiology use case

This section presents an agent-based model that describes the spreading of infectious diseases between humans. The model divides the population into three groups: susceptible, infected and recovered (SIR) agents. We compare our simulation results with the solution of the original SIR model from Kermack *et al.* (1927), which used the following three differential equations to describe the model dynamics: dS/dt=−βSI/N, dI/dt=βSI/N−γI and dR/dt=γI. *S*, *I* and *R* are the number of susceptible, infected and recovered individuals, *N* is the total number of individuals, *β* is the mean transmission rate and *γ* is the recovery rate.

**Fig. 6.**
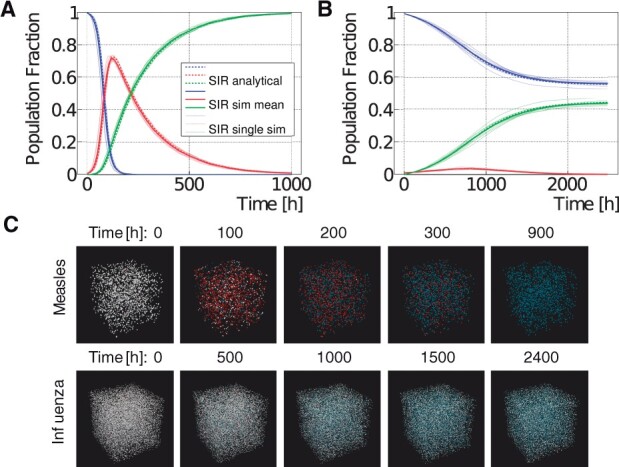
Measles and seasonal influenza SIR model results. (**A, B**) Comparison between agent-based (solid lines) and analytical (dashed lines) model for measles (A) and seasonal influenza (B). The agent-based simulation was repeated ten times. The individual simulation results are shown as thin solid lines. The bold solid line represents the mean from all simulations. The legend is shared between the two plots. (**C**) Visualization of the measles and influenza model for different time steps in 3D space. Susceptible persons are shown in white, infected persons in red, and recovered persons in blue

For our agent-based implementation (Figure 6C) we created a new agent (representing a person) that encompasses three new behaviors (see Figure 3). First, a susceptible agent became infected with the infection probability if an infected agent was within the infection radius. Second, an infected agent recovered with the recovery probability at every time step. Third, all agents moved randomly in space with toroidal boundary condition. The absolute distance an agent could travel in every time step was limited.

We selected two infectious diseases with different characteristics to verify our model: measles and seasonal influenza. We obtained values for the basic reproduction number *R*_0_ and recovery duration *T_R_* from the literature *(*Measles: $R_{0}=12.9$, *T_R_* = 8 days (Guerra *et al.*, 2017; World Health Organization, 2020), Influenza: $R_{0}=1.3,\;T_{R}=4.1$ days (Chowell *et al.*, 2008)) and determined the parameters *β* and *γ* for the analytical model, based on $R_{0}=\beta /\gamma$ and $\gamma =1/T_{R}$. For the agent-based model we set the recovery probability to *γ*, and determined the remaining parameters (infection radius, infection probability and maximum movement in one time step) using particle swarm optimization (Kennedy and Eberhart, 1995). Figure 6 shows that the agent-based model is in excellent agreement with the equation-based approach from (Kermack *et al.*, 1927) for measles and influenza.

Model specification required 181 lines of C++ code.

### 3.4 Performance

**Fig. 7.**
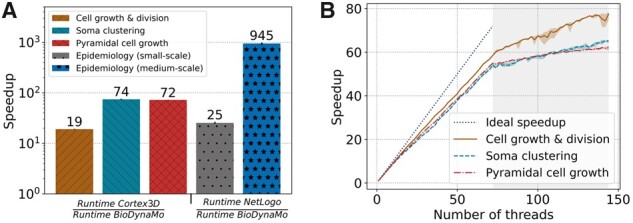
BioDynaMo performance analysis. (**A**) Speedup of BioDynaMo compared to the serial simulation platforms Cortex3D and NetLogo. Simulations use one CPU core except for the ‘epidemiology (medium-scale)’ benchmark, for which all 72 physical cores were available. The comparison with NetLogo uses the same simulation with different numbers of agents. Cortex3D supports only small-scale simulations. (**B**) Strong scaling behavior of BioDynaMo on a server with 72 physical cores, two threads per core and four NUMA domains. The gray area highlights hyper-threads

First, to demonstrate the performance improvements against established ABS platforms, we compared BioDynaMo with Cortex3D and NetLogo. Figure 7A shows the speedup of BioDynaMo for four simulations. We define speedup as the runtime of the compared ABS platform over the runtime of BioDynaMo. We observed a significant speedup between 19 and 74× for Cortex3D and 25× for NetLogo. The speedup was larger, when the simulation was more dynamic or more complex. Note that we set the number of threads available to BioDynaMo to one since Cortex3D and NetLogo are not parallelized. In the ‘epidemiology (medium-scale)’ benchmark we increased the number of available physical CPU cores to 72 and observe a three order of magnitude speedup of 945×. This result clearly shows the impact of parallelization on the overall performance. Although NetLogo is not parallelized, it benefits from parallel garbage collection. We could not perform a medium-scale analysis with Cortex3D, because it only supports simulations with a small number of agents.

Second, to evaluate the scalability of BioDynaMo, we measured the simulation time with an increasing number of threads. We increased the number of agents used in the comparison with Cortex3D and reduced the number of simulation timesteps to 10. Figure 7B shows the strong scaling analysis. We define the term ‘strong scaling’ as the property of a simulation platform to reduce the runtime of a simulation with a fixed size *x* with an increasing number of CPU cores *c*: $\mathrm{speedup}(c,x)=\frac{\mathrm{time}(1,x)}{\mathrm{time}(c,x)}$ (Hill, 1990).

All simulation parameters were kept constant, and the number of threads was increased from one to the number of logical cores provided by the benchmark server. The maximum speedup ranged between 62× and 77×, which corresponds to a parallel efficiency of 0.86 and 1.07. Performance improved even after all physical cores were utilized and hyper-threads were used. Hyper-threads are highlighted in gray in Figure 7B. We want to emphasize that even the pyramidal cell growth benchmark scaled well, despite the challenges of synchronization and load imbalance.

Third, we evaluated the impact of calculating the mechanical forces on the GPU using the cell growth and division, and soma clustering simulations. We excluded the pyramidal cell growth simulation because the current GPU kernel does not yet support cylinder geometry. The benchmarks were executed on System C (see Supplementary File SF1 Table 4), comparing an NVidia Tesla V100 GPU with 32 CPU cores (64 threads). We observed a speedup of 1.01× for cell growth and division, and 4.16× for soma clustering. The speedup correlated with the number of collisions in the simulation. The computational intensity is directly linked with the number of collisions between agents.

In summary, in the scalability test, we observed a minimum speedup of 62×. Furthermore, we measured a minimum speedup of 19× comparing BioDynaMo with Cortex3D both using a single thread. Based on these two observations, we conclude that on System A (see Supplementary File SF1 Table 4) BioDynaMo is more than three orders of magnitude faster than Cortex3D. In the comparison with NetLogo we observed a 945× speedup directly.

Based on these speedups, we executed the neuroscience, oncology and epidemiology use cases with one billion agents. Using all 72 physical CPUs on System B (see Supplementary File SF1 Table 4), we measured a runtime of 1 h 26 min, 6 h 22 min and 2 h, respectively. One billion agents, however, are not the limit. The maximum depends on the available memory and accepted execution duration. To be consistent across all use cases and keep our pipeline’s total execution time better manageable, we decided to run these benchmarks with one billion agents. Table 1 shows that available memory would permit an epidemiological and neuroscience simulation with two billion agents. With enough memory, BioDynaMo is capable of supporting hundreds of billions of agents.

## 4 Discussion

This article presented BioDynaMo, a novel open-source platform for agent-based simulations. Its modular software architecture allows researchers to implement models of distinctly different fields, of which neuroscience, oncology and epidemiology were demonstrated in this article. Although the implemented models follow a simplistic set of rules, the results that emerge from the simulations are prominent and highlight BioDynaMo’s capabilities. We do not claim that these models are novel, but we rather want to emphasize that BioDynaMo enables scientists to (i) develop models in various computational biology fields in a modular fashion, (ii) obtain results rapidly with the parallelized execution engine, (iii) scale up the model to billions of agents on a single server and (iv) produce results that are in agreement with validated experimental data. Although BioDynaMo is modular, we currently offer a limited number of ready-to-use simulation primitives. We are currently expanding our library of agents and behaviors to facilitate model development beyond the current capacity.

Ongoing work uses BioDynaMo to gain insights into retinal development, cryopreservation, multiscale (organ-to-cell) cancer modeling, radiation-induced tissue damage and more. Further efforts focus on accelerating drug development by replacing in vitro experiments with *in silico* simulations using BioDynaMo.

Our performance analysis showed improvements of up to three orders of magnitude over state-of-the-art baseline simulation software, allowing us to scale up simulations to an unprecedented number of agents. To the best of our knowledge, BioDynaMo is the first scalable ABS platform for neural development that scales to more than one billion agents. The same principles used to model axons and dendrites in the neuroscience use case could also be applied to simulate blood and lymphatic vessels.

We envision BioDynaMo to become a valuable tool in computational biology, fostering faster and easier simulation of complex and large-scale systems, interdisciplinary collaboration and scientific reproducibility.

## Funding

This work was supported by the CERN Knowledge Transfer office [to L.B. and A.H.]; the Israeli Innovation Authority [to A.H.]; the Research Excellence Academy from the Faculty of Medical Science of the Newcastle University [to J.dM.]; the UCY StartUp Grant scheme [to V.V.]; the Medical Research Council of the United Kingdom [MR/N015037/1 to R.B., MR/T004347/1 to M.K.]; the Engineering and Physical Sciences Research Council of the UK [EP/S001433/1 to R.B., NS/A000026/1, EP/N031962/1 to M.K.]; a PhD studentship funded by Newcastle University’s School of Computing [to J.J.]; the Wellcome Trust [102037 to M.K.]; the Guangci Professorship Program of Ruijin Hospital (Shanghai Jiao Tong Univ.) [to M.K.]; and by several donations by SAFARI Research Group’s industrial partners including Huawei, Intel, Microsoft, and VMware [to O.M.].

*Conflict of Interest*: none declared.

## Supplementary Material

btab649_Supplementary_Data
