# Supplementary material for: BioDynaMo: a modular platform for high-performance agent-based simulation
Source: Bioinformatics. 2021 Sep 16;38(2):453–60. doi: 10.1093/bioinformatics/btab649 (PMC8723141; doi:10.1093/bioinformatics/btab649)
Supplement: btab649_Supplementary_Data [file btab649_supplementary_data.zip › SF1-supplementary-materials.pdf]

Supplementary Materials

This document provides additional information about the design and implementation of BioDynaMo, use cases, and performance results. Furthermore, future directions and supplementary files are explained.

1 Design and implementation

Figure 2 in the main manuscript introduced the different abstraction layers of BioDynaMo. The following sections describe the missing low- and high-level features, and provide more details about the model building blocks.

1.1 Low-level features

1.1.1 Visualization

BioDynaMo currently uses ParaView (Ahrens *et al.*, 2005) as a visualization engine. There are two visualization modes, which we refer to as live mode and export mode. With live mode, the simulation can be visualized during runtime, whereas with export mode, the visualization state is exported to file and can only be visualized post-simulation. Live mode is a convenient approach to debug a simulation visually while it is executed. However, this can slow down the simulation considerably if used continuously. In export mode, the visualization state can be loaded by the visualization package for post-simulation processing (slicing, clipping, rendering, animating, etc.). BioDynaMo can visualize substance concentrations and gradients (see Figure 2), and the geometry of the supported agents.

Furthermore, it is possible to export any agent's data members. This information can then be used as input to ParaView filters, e.g., to highlight elements based on a specific property. The export of additional data members was used in Figure 2, for example, to color cells by their cell type.

1.1.2 BioDynaMo notebooks

Jupyter notebooks (Kluyver *et al.*, 2016) is a widely used web application to quickly prototype or demonstrate features of a software library. With notebooks, it is possible to easily create a website with inline code snippets that can be executed on-the-fly. ROOT expands these notebooks by offering a C++ backend in addition to the default Python backend. This allows us to provide a web interface to easily and quickly get started with BioDynaMo. Users do not need to install any software packages; a recent web browser is enough. It is also a convenient tool to interactively go through a demo or tutorial, which opens up possibilities to use BioDynaMo for educational purposes. BioDynaMo is the first agent-based simulation platform written in C++ that offers such an interface. BioDynaMo notebooks have already been successfully used to demonstrate and teach about pyramidal cell growth and were well-received by high-school students and teachers during CERN's official teachers and students programs. Figure 1 shows an example of how a BioDynaMo notebook looks. This example gives a brief introduction to pyramidal cells and follows up with a step-by-step explanation of how to simulate their growth with BioDynaMo. Interactive visualizations in the browser give users quick feedback about the simulation status. Lastly, tutorials written as BioDynaMo notebooks can be executed as part of our continuous integration pipeline and ensure that documentation stays in sync with the codebase. In Supplementary Tutorial ST01—ST15 we use this feature to explain BioDynaMo to new users.

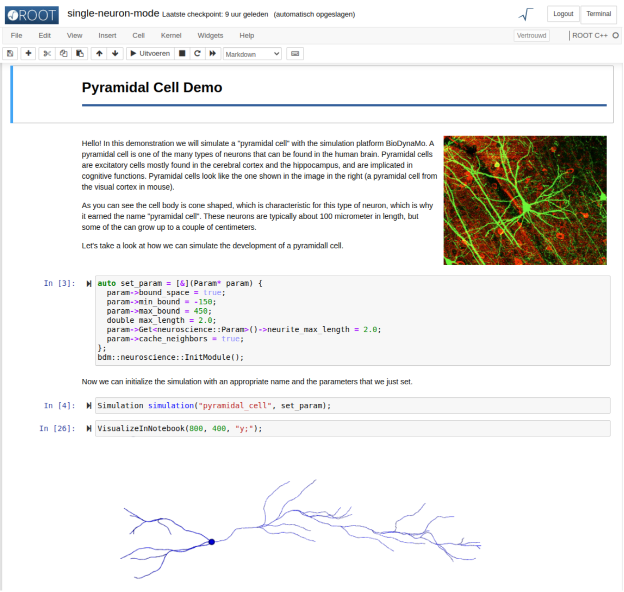

Fig. 1. BioDynaMo notebook. A convenient web interface to create and run simulations in a step-by-step manner. The inlining of text and media makes it possible to provide extra information. A few intermediary blocks have been removed to fit the final simulation output on the screenshot.

1.1.3 Backup and restore

BioDynaMo uses ROOT to integrate the backup and restore functionality transparently. This allows system failures to occur without losing valuable simulation data. Without any user intervention, all simulation data can be persisted to disk as system-independent binary files, called ROOT files, and restored into memory after a failure occurs. The ROOT file format is well-established and is the primary format for storing large quantities (petabytes) of data in high-energy physics experiments, such as CERN. To enable the backup and restore feature in BioDynaMo, one must simply specify the file name of the backup file. Additionally, one can set the interval at which a backup is performed. A low interval value ensures a low amount of data loss whenever a failure occurs, but also increases the incurred overhead for creating the backup files. The advised backup interval depends on the duration of the simulation.

1.1.4 Software quality assurance

Compromising on software quality can have severe consequences that can culminate in the retraction of published manuscripts (Miller, 2006). Therefore, we put tremendous effort into establishing a rigorous development workflow that follows industry best practices. Test-driven development—a practice from agile development (Beck and Gamma, 2000)—is at the core of our solution. BioDynaMo has over 400 tests distributed among unit, convergence, system, and installation tests. We monitor test coverage of our unit tests with the tool kcov (Kagstrom, 2020), and currently cover 79.8% lines of code. For each change to our repository (https://github.com/BioDynaMo/biodynamo), GitHub Actions (https://github.com/features/actions) executes the entire test suite and, upon success, updates the documentation on our website. Installation tests are executed on each supported operating system and ensure that all demo simulations run on a default system.

1.2 High-level features

1.2.1 Dynamic scheduling

In BioDynaMo the code that will be executed is controlled by behaviors and operations. Behaviors can be attached to individual agents and thus

allow very fine-grained control. Agent operations are usually executed for all agents (if no agent filters are specified). Both behaviors and operations can be created, added, removed, and destroyed during a simulation. This feature gives the user maximum flexibility to change the executed simulation code over time (Supplementary Tutorial ST13).

1.2.2 Parameter management

BioDynaMo simplifies the definition of simulation parameters by liberating the user from the burden to write code to parse parameter files or command line arguments.

1.2.3 Parameter optimization

The epidemiology use case presented in the results section of the main manuscript demonstrates how to define an experiment comprised of multiple input parameters, and a user-defined error function. This experiment definition is used in conjunction with the optimlib library (https://www.kthohr.com/optimlib.html) to determine model parameters that match the ground truth.

1.2.4 Space boundary conditions

BioDynaMo support three boundary conditions: (i) open, where the simulation space grows to encapsulate all agents in the simulation, (ii) closed, where artificial walls prevent agents from exiting the simulation space, and (iii) toroidal, where agents that leave the space on one side, will enter on the opposite side.

1.3 Model building blocks

In this section, we provide more details about the biological model BioDynaMo currently implements. This model closely resembles the principles from Cortex3D (Zubler and Douglas, 2009), but can be extended or replaced easily. Supplementary Tutorial ST15 for example demonstrates how to replace the default mechanical force implementation with a user-defined one. Table 1 lists the current agents, behaviors, and operations that the current BioDynaMo installation contains.

1.3.1 Mechanical forces

Growing realistic cell and tissue morphologies requires the consideration of mechanical interactions between agents. Therefore, BioDynaMo examines if two agents collide with each other at every timestep. To find all possible collisions, it is sufficient to evaluate neighbors in the environment. Whenever two agents (e.g. a cell body or a neurite element) overlap, a collision occurs. If a collision is detected, the engine calculates the mechanical forces that act on them.

The mechanical force calculation between spheres and cylinders follows the same approach as the implementation in Cortex3D (Zubler and Douglas, 2009). Both in BioDynaMo and Cortex3D, the magnitude of the force is computed based on (Pattana, 2006) and comprises a repulsive and attractive component:

$$F_N = k\delta - \gamma\sqrt{r\delta} \tag{1}$$

where δ indicates the spatial overlap between the two elements, and r denotes a combined measure of the two radii:

$$r = \frac{r_1 r_2}{r_1 + r_2} \tag{2}$$

where the radii denote the radii of the interacting spheres or cylinder.

Eq 1 comprises the effects of the structural tension from the pressure between the colliding membrane segments, and the attractive force due to the cell adhesion molecules. The magnitudes of these two force components depend upon the modifiable parameters k and γ. In the current form, as in Cortex3D, these are set to 2 and 1, respectively. After the forces have been determined, the agents change their 3D location depending on the force resulting from all the mechanical interactions with neighbors. More details about the implementation of the mechanical force, including the force between neighboring neurite elements, can be found in (Zubler and Douglas, 2009).

1.3.2 Extracellular diffusion

Signaling molecules, which differentiate and regulate cells, reach their destination through diffusion (Gurdon and Bourillot, 2001). A well-studied example of this process, called morphogen gradients, is the determination of vein positions in the wing of Drosophila (Bosch *et al.*, 2017).

BioDynaMo solves the partial differential equations that model the diffusion of extracellular substances (Fick's second law) with the discrete central difference scheme (Smith *et al.*, 1985). A grid with a variable resolution is imposed on the simulation space. At each timestep, the concentration value of each grid point is updated according to

$$\begin{aligned} u^{n+1}_{i,j,k} = \big(u^n_{i,j,k} &+ \frac{\nu\Delta t}{\Delta x^2}(u^n_{i+1,j,k} - 2u^n_{i,j,k} + u^n_{i-1,j,k}) \\ &+ \frac{\nu\Delta t}{\Delta y^2}(u^n_{i,j+1,k} - 2u^n_{i,j,k} + u^n_{i,j-1,k}) \\ &+ \frac{\nu\Delta t}{\Delta z^2}(u^n_{i,j,k+1} - 2u^n_{i,j,k} + u^n_{i,j,k-1})\big) \times (1-\mu)\,, \end{aligned} \tag{3}$$

where $u^n_{i,j,k}$ is the concentration value on grid point (i, j, k) at timestep n, ν is the diffusion coefficient, μ is the decay constant, Δt is the duration of one timestep, and Δx, Δy, and Δz are the distances between grid points in the x, y, and z direction, respectively. The distances between the grid points are inversely proportional to the resolution and determine the accuracy of the solver.

In BioDynaMo, it is possible to define the diffusion behavior at the simulation boundaries. In the default implementation, which we use for our examples in the result section, substances diffuse out of the simulation space.

In some cases, it is necessary to initialize substance concentrations artificially to simplify a simulation. Therefore, BioDynaMo provides predefined substance initializers (e.g. Gaussian) and accepts user-defined functions for arbitrary distributions. We used this functionality, for example, in the pyramidal cell growth simulation.

1.4 Code examples

In the main manuscript, Figure 3 depicts in an abstract way that BioDynaMo's software design is open for extension. With the three code examples in Listing 1 to 4—taken directly from the presented use cases and benchmarks—we want to emphasize how little code is required to add new functionality.

```
struct SimParam : public ParamGroup {
  BDM_PARAM_GROUP_HEADER(SimParam, 1);
  uint64_t cells_per_dim = 30;
  uint64_t iterations = 100;
};
```

Listing 1: Additional simulation parameters for the cell growth and division benchmark.

Table 1. **List of agents, events, and operations that BioDynaMo currently provides.**

	Description
**Agents**	
Agent	Agent is the base class for all agents in BioDynaMo. This class has a unique id that remains constant during the whole simulation and a collection of behaviors that have been attached to this agent. Agent contains functions to manage behaviors, and to remove itself from the simulation.
SphericalAgent	SphericalAgent extends Agent and adds a spherical agent geometry.
Cell	Cell extends Agent and represents a generic cell with a spherical shape. It includes attributes to describe its geometry, density, and adherence. Cell provides member functions to change its volume, move it in space, calculate mechanical forces, and divide it into two daughter cells. This cell division function creates a new daughter cell and distributes the volume of the mother cell according to the volume ratio parameter. The position of the daughter cell is determined based on the division axis.
NeuronSoma	NeuronSoma extends Cell. This class represents the cell body of a neuron and, like Cell, has a spherical shape. NeuronSoma has a list of neurite elements that extend from the cell body together with their attachment points. NeuronSoma adds a function to extend a new neurite element from the soma. This function takes two parameters: the diameter of the new neurite element, and the orientation of the cylinder in spherical coordinates. Zubler and Douglas (2009) contains more details.
NeuriteElement	NeuriteElement extends Agent and has a cylindrical shape with a proximal and distal end. A dendrite is modeled as a binary tree of neurite elements that are internally connected with springs to transmit forces to its proximal connection. The proximal connection can be a NeuriteSoma or a NeuriteElement. This class contains attributes to describe the cylindrical geometry, the spring, pointers to its proximal and distal connections, density, and adherence. A NeuriteElement can elongate, retract, split, branch, bifurcate, and extend a new side neurite. The following list describes these functions in more detail.<br>• *Split neurite element.* This function splits a neurite element into two segments. The neurite element whose split neurite element function was called becomes the distal one. The new neurite element will be the proximal one.<br>• *Extend side neurite.* This function adds a side neurite, if one of the distal connections is empty.<br>• *Bifurcate.* This function creates two new neurite elements and assigns them to the distal connections. This function can only be called for terminal neurite segments because both connections must be empty.<br>• *Branch.* This function splits the current neurite element into two elements and adds a new side branch at the proximal segment. It is, therefore, a combination of split neurite element and extend side neurite.<br>Zubler and Douglas (2009) contains more details.
**Behaviors**	
Chemotaxis	This behavior moves agents along the diffusion gradient (from low concentration to high).
Secretion	This behavior increases the substance concentration at the position of the agent.
GrowthDivision	This behavior grows cells to a specific size and divides them if they exceed the threshold.
GeneRegulation	This behavior calculates protein concentrations which are defined as differential equations.
StatelessBehavior	This behavior reduces the amount of code that has to be written for behaviors without attributes.
**Agent operations**	
BehaviorOp	This operation runs all behaviors which are attached to the agent.
BoundSpaceOp	This operation enforces the space boundary condition (see Section 1.2.4) which can be open, closed or toroidal.
DiscretizationOp	This operation calls the agent's discretization function. NeuriteElement uses this function to split itself if it becomes too long, or merge with another segment if it is too short.
MechanicalForcesOp	This operation calls the agent's calculate displacement function and moves the agent accordingly. The calculate displacement function contains the implementation how an agent moves based on all forces that act on it.
**Standalone operations**	
DiffusionOp	This operation calls the update function of all substances in the simulation.
UpdateEnvironmentOp	This operation calls the update function of the environment algorithm.
UpdateTimeSeriesOp	This operation calls the update function of the TimeSeries object which collects relevant data from the current iteration which can be analysed at the end of the simulation.
VisualizationOp	This operation updates the live visualization or generates visualization files for later analysis.

2 Results

2.1 Use cases

The behavior governing apical and basal dendrite growth (neuroscience use case) is outlined in Algorithm 1. Algorithm 2 shows pseudocode for the tumor cell behavior as used in the oncology use case. The epidemiology use case adds three new behaviors which are depicted in Algorithm 3 (infection), Algorithm 4 (recovery), and Algorithm 5 (random movement).

```
/// Possible states.
enum State { kSusceptible, kInfected, kRecovered };

class Person : public Cell {
  BDM_AGENT_HEADER(Person, Cell, 1);

 public:
  Person() {}
  explicit Person(const Double3& position) : Base(position) {}
  virtual ~Person() {}

  /// This attribute stores the current state of the person.
  int state_ = State::kSusceptible;
};
```

Listing 2: New agent class used in the epidemiology use case.

```
struct Recovery : public Behavior {
  BDM_BEHAVIOR_HEADER(Recovery, Behavior, 1);

  Recovery() {}

  void Run(Agent* a) override {
    auto* person = bdm_static_cast<Person*>(a);
    if (person->state_ == kInfected) {
      auto* sim = Simulation::GetActive();
      auto* random = sim->GetRandom();
      auto* sparam = sim->GetParam()->Get<SimParam>();
      if (random->Uniform(0, 1) <= sparam->recovery_probability) {
        person->state_ = State::kRecovered;
      }
    }
  }
};
```

Listing 3: New behavior used in the epidemiology use case.

Model parameters can be found in Table 2 for the neuroscience use case, Table 3 for the oncology use case, and Table 4 for the epidemiology use case.

2.2 Performance

In this section, we provide additional information about our performance evaluation. Table 5 details the experimental setup that we used for our benchmarks and Table 6 lists the measured runtime and memory consumptions on these systems.

Cell growth and division benchmark. The starting condition of this simulation was a 3D grid of cells. These cells were programmed to grow to a specific diameter and divide afterward. This simulation had high cell density and slow-moving cells. This simulation covered mechanical interaction between spherical cells, biological behavior, and cell division.

Soma clustering benchmark. The goal of this model was to cluster two types of cells that are initially randomly distributed. These cells are represented in red and blue in Figure 2A and B. Each cell type secreted a specific extracellular substance which attracted homotypic cells. Substances diffused through the extracellular matrix following Eq 3. We modeled cell processes with two behaviors, ran in sequence: substance secretion (Algorithm 6) and chemotaxis (Algorithm 7). We set the parameter `secretion_quantity` to 1 and `gradient_weight` to 0.75. During the simulation, cell clusters formed depending on their type. The final simulation state after 6000 time steps is shown in Figure 2B. Clusters were associated with non-homogeneous extracellular substance distributions, as shown in Figure 2C and Figure 3. Besides being used as a benchmark, this example demonstrates the applicability of BioDynaMo for modeling biological systems, including the dynamics of chemicals such as oxygen or growth factors. The simulation consisted of 68 lines of C++ code. Table 6 shows the performance on different systems. There are three main differences comparing this simulation with the previous cell

```
// File: pyramidal_cell.h
#ifndef PYRAMIDAL_CELL_H_
#define PYRAMIDAL_CELL_H_

#include "biodynamo.h"
#include "neuroscience/neuroscience.h"

namespace bdm {

enum Substances { kApical, kBasal };

struct ApicalDendriteGrowth : public Behavior {
  BDM_BEHAVIOR_HEADER(ApicalDendriteGrowth, Behavior, 1);
  ApicalDendriteGrowth() { AlwaysCopyToNew(); }
  virtual ~ApicalDendriteGrowth() {}
  void Initialize(const NewAgentEvent& event) override {
    Base::Initialize(event);
    can_branch_ = false;
  }
  void Run(Agent* agent) override { /* omitted */ }
 private:
  bool init_ = false;
  bool can_branch_ = true;
  DiffusionGrid* dg_guide_ = nullptr;
};

struct BasalDendriteGrowth : public Behavior {
  BDM_BEHAVIOR_HEADER(BasalDendriteGrowth, Behavior, 1);
  BasalDendriteGrowth() { AlwaysCopyToNew(); }
  virtual ~BasalDendriteGrowth() {}
  void Run(Agent* agent) override { /* omitted */ }
 private:
  bool init_ = false;
  DiffusionGrid* dg_guide_ = nullptr;
};

inline void AddInitialNeuron(const Double3& position) {
  auto* soma = new neuroscience::NeuronSoma(position);
  soma->SetDiameter(10);
  Simulation::GetActive()->GetResourceManager()->AddAgent(soma);

  auto* apical_dendrite = soma->ExtendNewNeurite({0, 0, 1});
  auto* basal_dendrite1 = soma->ExtendNewNeurite({0, 0, -1});
  auto* basal_dendrite2 = soma->ExtendNewNeurite({0, 0.6, -0.8});
  auto* basal_dendrite3 = soma->ExtendNewNeurite({0.3, -0.6, -0.8});

  apical_dendrite->AddBehavior(new ApicalDendriteGrowth());
  basal_dendrite1->AddBehavior(new BasalDendriteGrowth());
  basal_dendrite2->AddBehavior(new BasalDendriteGrowth());
  basal_dendrite3->AddBehavior(new BasalDendriteGrowth());
}

/// Create and initialize substances for neurite attraction
inline void CreateExtracellularSubstances(const Param* p) {
  using MI = ModelInitializer;
  MI::DefineSubstance(kApical, "substance_apical", 0, 0,
                      p->max_bound / 80);
  MI::DefineSubstance(kBasal, "substance_basal", 0, 0,
                      p->max_bound / 80);
  // initialize substance with gaussian distribution
  auto a_initializer = GaussianBand(p->max_bound, 200, Axis::kZAxis);
  auto b_initializer = GaussianBand(p->min_bound, 200, Axis::kZAxis);
  MI::InitializeSubstance(kApical, a_initializer);
  MI::InitializeSubstance(kBasal, b_initializer);
}

inline int Simulate(int argc, const char** argv) {
  neuroscience::InitModule();
  Simulation simulation(argc, argv);
  AddInitialNeuron({150, 150, 0});
  CreateExtracellularSubstances(simulation.GetParam());
  simulation.GetScheduler()->Simulate(500);
  return 0;
}

} // namespace bdm
#endif // PYRAMIDAL_CELL_H_
// ----------------------------------------------------------------------
// File: pyramidal_cell.cc
#include "pyramidal_cell.h"
int main(int c, const char** v) { return bdm::Simulate(c, v); }
```

Listing 4: **Pyramidal cell growth simulation code**. This example shows the required C++ code to simulate the growth of a single pyramidal cell as shown in Figure 4A in the main manuscript. All classes and functions which are not defined in this example are provided by BioDynaMo. Only the body of the two behavior's `Run` methods has been ommited, but are provided in Supplementary File S3 and Algorithm 1.

growth and division simulation. First, this simulation covered extracellular diffusion. Second, cells moved more rapidly. Third, the number of cells remained constant during the simulation.

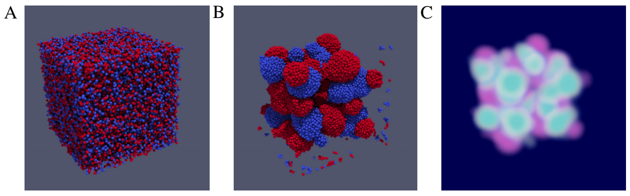

Fig. 2. Soma clustering simulation. This simulation contains two types of cells and two extracellular substances. Each cell secretes a substance and moves into the direction of the substance gradient. Cells are distributed randomly in the beginning (A) and form clusters during the simulation. (B) Cell clusters at the end of the simulation. (C) Substance concentrations at the end of the simulation. A video is available at SV4-soma-clustering.mp4.

Pyramidal cell growth benchmark. We used the pyramidal cell model from the neuroscience use case as a building block (see Figure 4 in the main manuscript). The simulation started with a 2D grid of initial neurons on the z-plane and started growing them. This simulation has three distinctive features. First, activity was limited to a neurite growth front, while the rest of the simulation remained static. This introduced a load imbalance for parallel execution. Second, the neurite implementation modified neighboring agents. Hence, synchronization was required between multiple threads to ensure correctness. Third, the simulation had only static substances, i.e., substance concentrations and gradients did not change over time.

3 Availability and future directions

BioDynaMo is an open-source project under the Apache 2.0 license and can be found on Github (https://github.com/BioDynaMo/biodynamo). The documentation is split into three parts: API reference, user guide, and developer guide. Furthermore, a Slack channel is available for requesting assistance or guidance from the BioDynaMo development team.

BioDynaMo officially supports the following operating systems: Ubuntu (18.04, 20.04), CentOS 7, and macOS (10.15, 11.1). We test BioDynaMo on these systems and provide prebuilt binaries for third party dependencies: ROOT and ParaView.

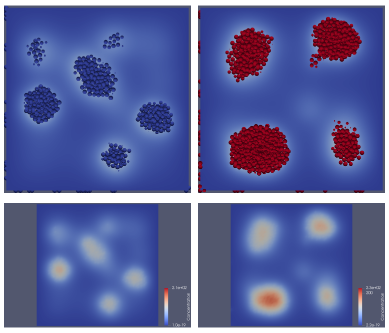

Fig. 3. Soma clustering cross section. Cell positions coincide with regions of high substance concentration. The first row shows substance concentrations and cells, while the second row shows substance concentrations only. Columns show cell type with the corresponding substance.

All of the results presented in the paper can be reproduced following the instructions in Supplementary Information.

By designing BioDynaMo in a modular and extensible way, we laid the foundation to create new functionalities easily. We encourage the life science community to contribute their developments back to the open-source codebase of BioDynaMo. Over time, the accumulation of all these contributions will form the BioDynaMo open-model library, as shown in Figure 4. This library will help scientists accelerate their research by providing the required building blocks (agents, biological behavior, etc.) for their simulation. Currently, we collect these contributions in our Github repository (https://github.com/BioDynaMo/biodynamo).

4 Supporting information

SF2-reproduce-results.md **Instructions on how to reproduce all results presented in this paper.**

SF3-code.tar.gz **Codebase to reproduce all results presented in this paper.** This file contains all code necessary to reproduce performance results, plots, visualizations, and videos shown in this paper. Furthermore, it contains more details about the hardware and software configuration of the different systems described in Table 5.

SF4-bdm-publication-image.tar.gz **Docker image to reproduce all results presented in this paper.** We provide a Docker image to simplify the process of executing our simulations and benchmarks and to guarantee long-term reproducibility. The only requirement that users must install is a recent version of the Docker engine. All other prerequisites are already provided in the ready-to-use, self-contained Docker image. This approach does not rely on content hosted somewhere on the internet that might become unavailable in the future.

SF5-raw-results.tar.gz **Raw results.** This archive contains all raw results from the simulations and benchmarks shown in this paper.

SV1-single-pyramidal-cell.mp4 **Single pyramidal cell growth simulation, as shown in Figure 4A in the main manuscript.**

SV2-large-scale-neuronal-development.mp4 **Large-scale pyramidal cell growth simulation, as shown in Figure 4C in the main manuscript.**

SV3-tumor-spheroid.mp4 **Tumor spheroid growth simulation, as shown in Figure 5B in the main manuscript.**

SV4-soma-clustering.mp4. **Soma clustering simulation, as shown in Figure 2.**

ST01—ST15.* **Fifteen tutorials to demonstrate various aspects of BioDynaMo.**

References

Ahrens, J., Geveci, B., and Law, C. (2005). ParaView: An End-User Tool for Large-Data Visualization. In *Visualization Handbook*, pages 717–731. Elsevier.

Beck, K. and Gamma, E. (2000). *Extreme programming explained: embrace change*. addison-wesley professional.

Bosch, P. S., Ziukaite, R., Alexandre, C., Basler, K., and Vincent, J.-P. (2017). Dpp controls growth and patterning in Drosophila wing precursors through distinct modes of action. *eLife*, **6**, e22546.

Gurdon, J. B. and Bourillot, P.-Y. (2001). Morphogen gradient interpretation. *Nature*, **413**(6858), 797–803.

Kagstrom, S. (2020). SimonKagstrom/kcov. original-date: 2010-08-23T12:03:31Z.

Kluyver, T., Ragan-Kelley, B., Pérez, F., Granger, B. E., Bussonnier, M., Frederic, J., Kelley, K., Hamrick, J. B., Grout, J., Corlay, S., *et al.* (2016). Jupyter notebooks-a publishing format for reproducible computational workflows. In *ELPUB*, pages 87–90.

Miller, G. (2006). A Scientist's Nightmare: Software Problem Leads to Five Retractions. *Science*, **314**(5807), 1856–1857.

Pattana, S. (2006). *Division d'un milieu cellulaire sous contraintes mécaniques: utilisation de la mécanique des matériaux granulaires*. Ph.D. thesis, Université Montpellier II, place Eugéne Bataillon 34095 Montpellier Cedex 5 France.

Smith, G., Smith, G., Smith, G., Smither, M., and Press, O. U. (1985). *Numerical Solution of Partial Differential Equations: Finite Difference Methods*. Oxford applied mathematics and computing science series. Clarendon Press.

Zubler, F. and Douglas, R. (2009). A framework for modeling the growth and development of neurons and networks. *Frontiers in computational neuroscience*, **3**, 25.

Algorithm 1: Apical and basal dendrite growth.

```
input: neurite, growth_factor, diameter_threshold,
       diameter_threshold_two, growth_speed,
       branching_probability, old_direction_weight,
       randomness_weight, gradient_weight, shrinkage,
       can_branch
diameter ← neurite.GetDiameter();
if diameter > diameter_threshold then
    old_direction ← neurite.GetDirection();
    pos ← neurite.GetPosition();
    gradient ←
      growth_factor.GetNormalizedGradient(pos);
    direction ← old_direction × old_direction_weight +
      gradient × gradient_weight + RandomUniform3(-1, 1) ×
      randomness_weight;
    neurite.Extend(growth_speed, direction);
    neurite.SetDiameter(diameter- shrinkage);
    if neurite.IsApical() then
        if can_branch and neurite.IsTerminal() and
          diameter < diameter_threshold_two and
          RandomUniform(0, 1) < branching_probability then
            branching_direction ←
              CalculateBranchingDirection(neurite);
            neurite.Branch(branching_direction);
        end
    end
    else if RandomUniform(0, 1) < branching_probability
      then
        neurite.Bifurcate();
    end
end
```

Table 2. **Model parameters for the pyramidal cell growth simulation.**

Parameter	Apical dendrite	Basal dendrite
Diameter threshold	0.575	0.75
Diameter threshold two	0.55	
Old direction weight	4	6
Gradient weight	0.06	0.03
Randomness weight	0.3	0.4
Growth speed	100	50
Shrinkage	0.00071	0.00085
Branching probability	0.038	0.006

Algorithm 2: Cancer cell behavior.

```
input: cell, minimum_cell_age, death_probability,
       displacement_rate, growth_speed, division_probability
random_vector ← RandomUniform3(-1, 1);
brownian ← random_vector ÷ random_vector.L2Norm();
cell.UpdatePosition(brownian × displacement_rate);
if age >= minimum_cell_age and
  RandomUniform(0, 1) < death_probability then
    cell.RemoveFromSimulation();
    return;
end
age ← age + 1;
if cell.GetDiameter < max_diameter then
    cell.IncreaseVolume(growth_speed);
else if RandomUniform(0, 1) < division_probability then
    cell.Divide();
end
```

Table 3. **Model parameters for the tumor spheroid growth simulations.**

Parameter [dimensions]	2000	4000 cells/well	8000
Cell growth rate [μm^3/h]	42.0	35.0	29.9
Minimum cell age to apoptosis [h]	87	87	87
Division probability	0.0215	0.0215	0.0215
Cell death probability	0.033	0.033	0.033
Maximum cell speed [μm/h]	1.0	0.9	0.2
Cell–ECM adherence coefficient [dimensionless]	1.8	1.8	1.8
Random cell movement = displacement rate [μm/h]	0.005	0.005	0.0005

Algorithm 3: Infection behavior.

```
input: person, environment, infection_probability,
       infection_radius
if person.GetState() == susceptible and
  RandomUniform(0,1) < infection_probability then
    neighbors
     ← environment.GetNeighbors(infection_radius);
    for each neighbor in neighbors do
        if neighbor.GetState() == infected then
            person.SetState(infected);
        end
    end
end
```

Algorithm 4: Recovery behavior.

```
input: person, recovery_probability
if person.GetState() == infected and
  RandomUniform(0,1) < recovery_probability then
    person.SetState(recovered);
end
```

Algorithm 5: Random movement behavior.

```
input: person, speed, max_bound
position ← person.GetPosition();
movement ← RandomUniform3(-1, 1).L2Norm();
new_position ← position + movement × speed;
for each el in new_position do
    el ← fMod(el, max_bound);
    if el < 0 then
        el ← max_bound + el;
    end
end
person.SetPosition(new_position);
```

Table 4. **Model parameters for the epidemiological use case.**

Parameter [dimension]	Measles	Seasonal Influenza
β (analytical solution)	0.06719	0.01321
γ (analytical solution)	0.00521	0.01016
Time step interval [h]	1	1
Number of time steps	1000	2500
Cubic simulation space length [m]	100	215
Initial number of susceptible persons	2000	20000
Initial number of infected persons	20	200
Infection radius [m]	3.24179	3.2123
Infection probability	0.28510	0.04980
Recovery probability	0.00521	0.01016
Max movement per time step [m]	5.78594	4.2942

Algorithm 6: Soma clustering substance secretion.

```
input: cell, diffusion_grid, secretion_quantity
pos ← cell.GetPosition();
diffusion_grid.IncreaseConcentrationBy(pos,
  secretion_quantity);
```

Algorithm 7: Soma clustering chemotaxis.

```
input: cell, diffusion_grid, gradient_weight
pos ← cell.GetPosition();
grad ← diffusion_grid.GetNormalizedGradient(pos);
cell.UpdatePosition(grad ×gradient_weight);
```

Table 5. **Experimental setup.** Main parameters of the systems that we used to run the benchmarks of this paper. SF3-code.tar.gz contains more details.

System	Main memory	CPU / GPU	OS
A	504 GB	Server with four Intel(R) Xeon(R) E7-8890 v3 CPUs @ 2.50GHz with a total of 72 physical cores, two threads per core and four NUMA nodes.	CentOS 7.9.2009
B	1008 GB		
C	191 GB	Server with two Intel(R) Xeon(R) Gold 6130 CPUs @ 2.10GHz with 16 physical cores, two threads per core, and two NUMA nodes.<br>One NVidia Tesla V100 SXM2 GPU with 32 GB memory.	CentOS 7.7.1908
D	16 GB	Dell Latitude 7480 Laptop from 2017.<br>One Intel(R) Core(TM) i7-7600U CPU @ 2.80GHz with two physical cores and two threads per core.<br>One Intel HD Graphics 620 GPU with 64 MB eDRAM.	Ubuntu 20.04.1 LTS

Table 6. **Performance data.** The values in column “Agents" and “Diffusion volumes" are taken from the end of the simulation. Runtime measures the wall-clock time to simulate the number of iterations. It excludes the time for simulation setup and visualization.

Simulation	Agents	Diffusion volumes	Iterations	System (Table 5)	Physical CPUs	Runtime	Memory
Neuroscience use case							
Single (Figure 4A in the main manuscript)	1 494	250	500	A	1	0.16 s	382 MB
				D	1	0.12 s	479 MB
Large-scale (Figure 4C in the main manuscript)	9 036 986	65 536	500	A	72	35 s	6.47 GB
				D	2	11 min 28 s	5.37 GB
Very-large-scale	1 018 644 154	5 606 442	500	B	72	1 h 24 min	438 GB
Oncology use case (Figure 5 in the main manuscript)							
2000 initial cells	4 177	0	312	A	1	1.05 s	382 MB
				D	1	0.832 s	480 MB
4000 initial cells	5 341	0	312	A	1	1.76 s	382 MB
				D	1	1.34 s	480 MB
8000 initial cells	7 861	0	288	A	1	3.27 s	384 MB
				D	1	2.60 s	482 MB
Large-scale	1 000 3925	0	288	A	72	1 min 42 s	7.42 GB
				D	2	43 min 56 s	5.84 GB
Very-large-scale	986 054 868	0	288	B	72	6 h 21 min	604 GB
Epidemiology use case (Figure 6C in the main manuscript)							
Measles	2 010	0	1000	A	1	0.53 s	381 MB
				D	1	0.42 s	479 MB
Seasonal Influenza	20 200	0	2500	A	1	16.41 s	383 MB
				D	1	16.40 s	479 GB
Medium-scale (measles)	100 500	0	1000	A	72	1.36 s	1 GB
Large-scale (measles)	10 050 000	0	1000	A	72	59.19 s	5.87 GB
				D	2	19 min 18 s	5.41 GB
Very-large-scale (measles)	1 005 000 000	0	1000	B	72	2 h 0 min	495 GB
Soma clustering (Figure 2)	32 000	1 240 000	6 000	A	72	12.91 s	1.02 GB
				D	2	2 min 7 s	522 MB

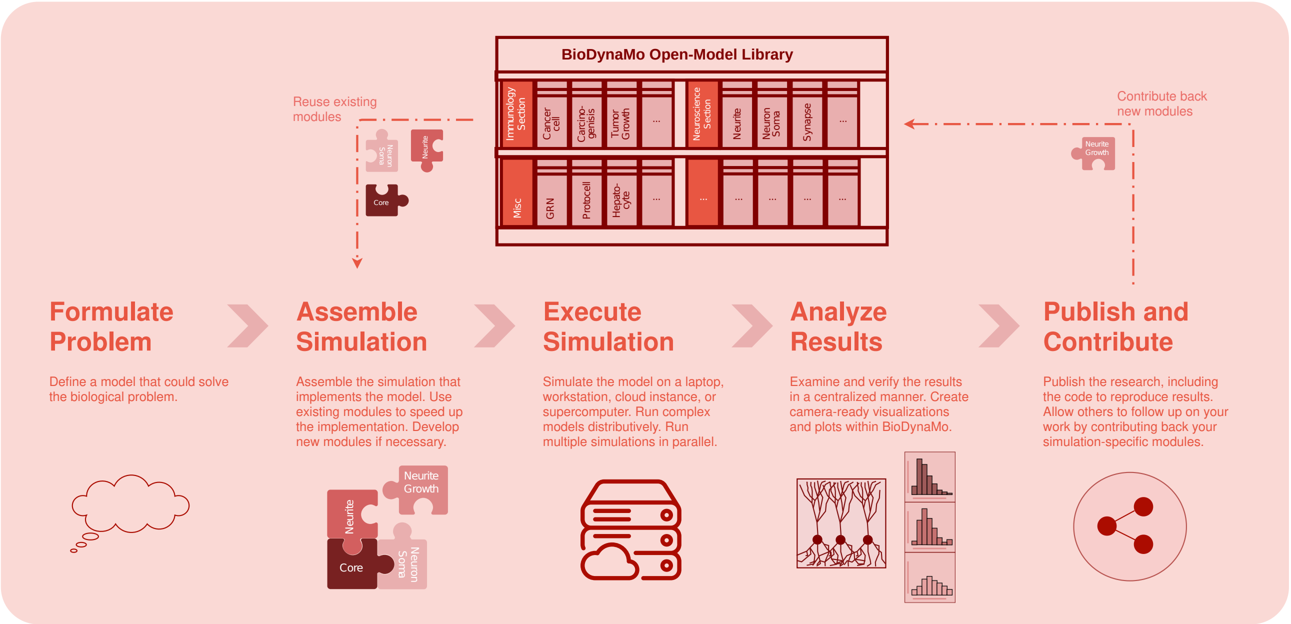

Fig. 4. BioDynaMo platform. Vision of BioDynaMo, a platform to accelerate in silico experiments.
