# Supplementary material for: BioDynaMo: a modular platform for high-performance agent-based simulation
Source: Bioinformatics. 2021 Sep 16;38(2):453–60. doi: 10.1093/bioinformatics/btab649 (PMC8723141; doi:10.1093/bioinformatics/btab649)

Create agents in 3D space

Author: Lukas Breitwieser
In this tutorial we want to demonstrate different functions to initialize agents in space.
Let's start by setting up BioDynaMo notebooks.

In [1]:

```
%jsroot on
gROOT->LoadMacro("${BDMSYS}/etc/rootlogon.C");
```

```
INFO: Created simulation object 'simulation' with UniqueName='simulati
on'.
```

We use `SphericalAgent` s with $diameter = 10$ for all consecutive examples.

In [2]:

```
auto create_agent = [](const Double3& position) {
  auto* agent = new SphericalAgent(position);
  agent->SetDiameter(10);
  return agent;
};
```

We define the number of agents that should be created for functions that require this parameter.

In [3]:

```
uint64_t num_agents = 300;
```

We define two helper functions that reset the simulation to the empty state and one to visualize the result.

In [4]:

```
void Clear() {
  simulation.GetResourceManager()->ClearAgents();
}
```

In [5]:

```
void Vis() {
  simulation.GetScheduler()->FinalizeInitialization();
  VisualizeInNotebook();
}
```

Create agents randomly inside a 3D cube

Cube: $x_{min} = y_{min} = z_{min} = -200$ and $x_{max} = y_{max} = z_{max} = 200$
By default a uniform random number distribution is used.

In [6]:

```
Clear();
ModelInitializer::CreateAgentsRandom(-200, 200, num_agents, create_agent);
Vis();
```

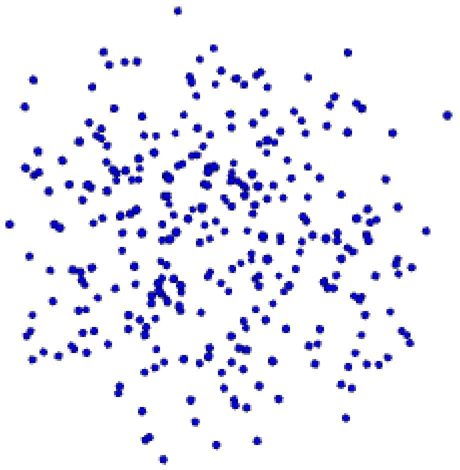

Create agents randomly inside a 3D cube using a gaussian distribution

Cube: $x_{min} = y_{min} = z_{min} = -200$ and $x_{max} = y_{max} = z_{max} = 200$
Gaussian: $\mu = 0$, $\sigma = 20$
Note the extra parameter rng passed to `CreateAgentsRandom`

In [7]:

```
Clear();
auto rng = simulation.GetRandom()->GetGausRng(0, 20);
ModelInitializer::CreateAgentsRandom(-200, 200, num_agents, create_agent, &rng);
Vis();
```

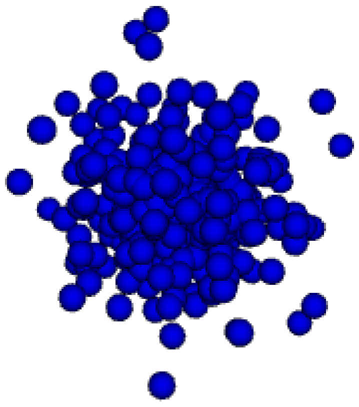

Create agents randomly inside a 3D cube using an exponential distribution

Cube: $x_{min} = y_{min} = z_{min} = -200$ and $x_{max} = y_{max} = z_{max} = 200$
Exponential: $\tau = 100$
Note the extra parameter rng passed to `CreateAgentsRandom`

In [8]:

```
Clear();
auto rng = simulation.GetRandom()->GetExpRng(100);
ModelInitializer::CreateAgentsRandom(-200, 200, num_agents, create_agent, &rng);
Vis();
```

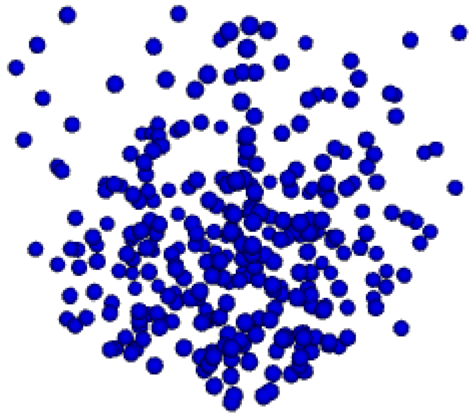

Create agents randomly inside a 3D cube using a 3D gaussian distribution

Cube: $x_{min} = y_{min} = z_{min} = -200$ and $x_{max} = y_{max} = z_{max} = 200$
3D gaussian: $\mu_x = \mu_y = \mu_z = 0$, $\sigma_x = 100$, $\sigma_y = 50$, $\sigma_z = 20$
The gaussian distribution we used earlier in this tutorial used the same parameters μ and σ for all three dimentions. In this example we want to use different values for σ in each dimension. Therefore we have to use a 3D guassian. Since BioDynaMo does not have a predefined 3D gaussian, we have to define the function ourselves.

In [9]:

```
Clear();
auto gaus3d = [](const double* x, const double* params) {
    auto mx = params[0];
    auto my = params[2];
    auto mz = params[4];
    auto sx = params[1];
    auto sy = params[3];
    auto sz = params[5];
    auto ret = (1.0/(sx * sy * sz *std::pow(2.0*Math::kPi, 3.0/2.0))) *
        std::exp(-std::pow(x[0] - mx, 2.0)/std::pow(sx, 2.0) -
                  std::pow(x[1] - my, 2.0)/std::pow(sy, 2.0) -
                  std::pow(x[2] - mz, 2.0)/std::pow(sz, 2.0));
    return ret;
};
auto* random = simulation.GetRandom();
auto rng = random->GetUserDefinedDistRng3D(gaus3d, {0, 100, 0, 50, 0, 20},
                                           -200, 200, -200, 200, -200, 200);
ModelInitializer::CreateAgentsRandom(-200, 200, num_agents, create_agent, &rng);
Vis();
```

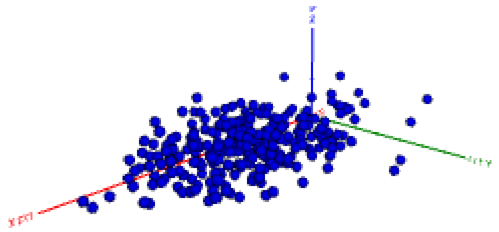

Create agents randomly on a sphere

Center of the sphere $0, 0, 0$
Radius: 100

In [10]:

```
Clear();
ModelInitializer::CreateAgentsOnSphereRndm({0, 0, 0}, 100, num_agents,
                                           create_agent);
Vis();
```

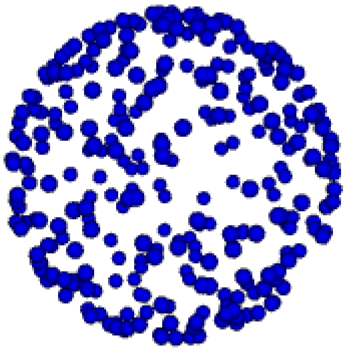

Create 3D grid of agents

Number of agents per dimension: 10

Space between agents: 20

With this parameters `Grid3D` will create 1000 agents.

In [11]:

```
Clear();
uint64_t agents_per_dim = 10;
double space_between_agents = 20;
ModelInitializer::Grid3D(10, 20, create_agent);
Vis();
```

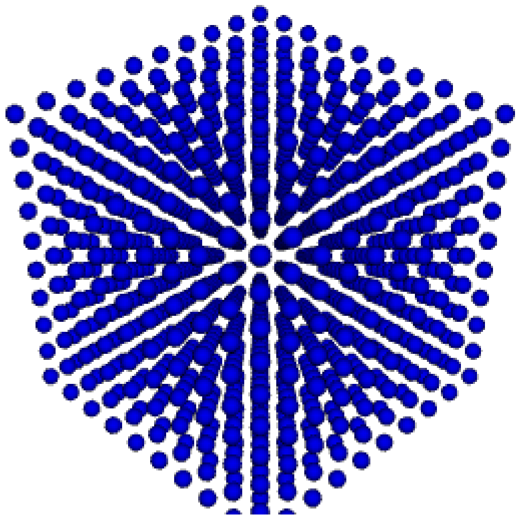

Create agents on a surface

We create agents between $x_{min} = y_{min} = -100$ and $x_{max} = y_{max} = 100$ with spacing of 10 beween agents. The z-coordinate is defined by the function $f(x, y) = 10 * sin(x/20) + 10 * sin(y/20)$

In [12]:

```
Clear();
auto f = [](const double* x, const double* params) {
    return 10 * std::sin(x[0] / 20.) + 10 * std::sin(x[1] / 20.0);
};
ModelInitializer::CreateAgentsOnSurface(f, {}, -100, 100, 10, -100, 100, 10,
                                        create_agent);
Vis();
```

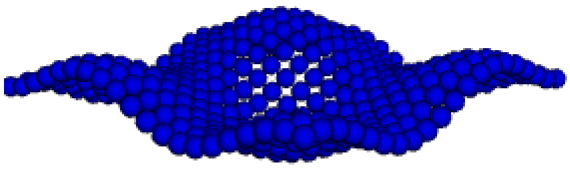

Create agents on a surface randomly

We use the same parameters as in the example before, but this time we want to place agents randomly on this surface. Therefore, x, and y coordinate are sampled from a uniform distribution between $x_{min} = y_{min} = -100$ and $x_{max} = y_{max} = 100$.

In [13]:

```
Clear();
ModelInitializer::CreateAgentsOnSurfaceRndm(f, {}, -100, 100, -100, 100, num_agents
                                            create_agent);
Vis();
```

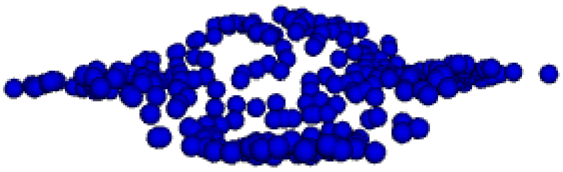

Supplement: btab649_Supplementary_Data [file btab649_supplementary_data.zip › ST01-model-initializer.pdf]
