# Supplementary material for: BioDynaMo: a modular platform for high-performance agent-based simulation
Source: Bioinformatics. 2021 Sep 16;38(2):453–60. doi: 10.1093/bioinformatics/btab649 (PMC8723141; doi:10.1093/bioinformatics/btab649)
Supplement: btab649_Supplementary_Data [file btab649_supplementary_data.zip › ST02-user-defined-random-number-distribution.pdf]

Generate random samples from a user-defined distribution

Author: Lukas Breitwieser

In this tutorial we demonstrate how to create a random number generator that draws samples from a user-defined distribution.

Let's start by setting up BioDynaMo notebooks

In [1]:

```
%jsroot on
gROOT->LoadMacro("${BDMSYS}/etc/rootlogon.C");
```

```
INFO: Created simulation object 'simulation' with UniqueName='simulati
on'.
```

We have to create a `TCanvas` object to draw results in this notebook.

In [2]:

```
TCanvas c("", "", 400, 300);
c.SetGrid();
```

Let's assume that we want to generate random numbers from a student-t distribution.
Class `Random` (https://biodynamo.org/api/classbdm_1_1Random.html) does not provide a direct function for that.
Therefore, we use the user-defined distribution feature `Random::GetUserDefinedDistRng1D` .
Fortunately, ROOT already provides a function called `tdistribution_pdf` that we can use.
Have a look at the following two links for more math functions: TMath (https://root.cern/doc/master/namespaceTMath.html) and statistical functions (https://root.cern/doc/master/group__StatFunc.html)

In [3]:

```
auto* random = simulation.GetRandom();
auto distribution = [](const double* x, const double* param) {
    return ROOT::Math::tdistribution_pdf(*x, 1.0);
};
auto udd_rng = random->GetUserDefinedDistRng1D(distribution, {}, -5, 10);
```

The returned random number generator has a function to draw the distribution.

In [4]:

```
udd_rng.Draw();
c.Draw();
```

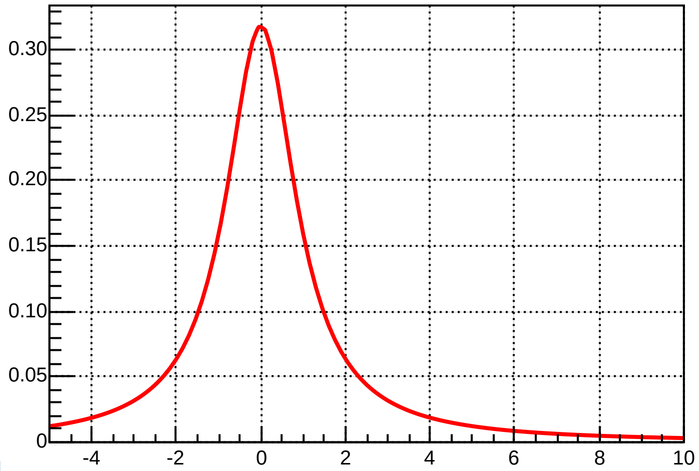

In the next step we want to verify that the created random number generator follows the specified distribution. Therefore, we create a histogram with 100 bins in the range [-5, 10] and fill it with 10000 samples.

In [5]:

```
TH1F h("","", 100, -5, 10);
for (int i = 0; i < 10000; ++i){
    auto rndm_sample = udd_rng.Sample();
    h.Fill(rndm_sample);
}
```

Let's draw the result:

In [6]:

```
h.SetFillColor(kBlue-10);
h.Draw();
c.Draw();
```

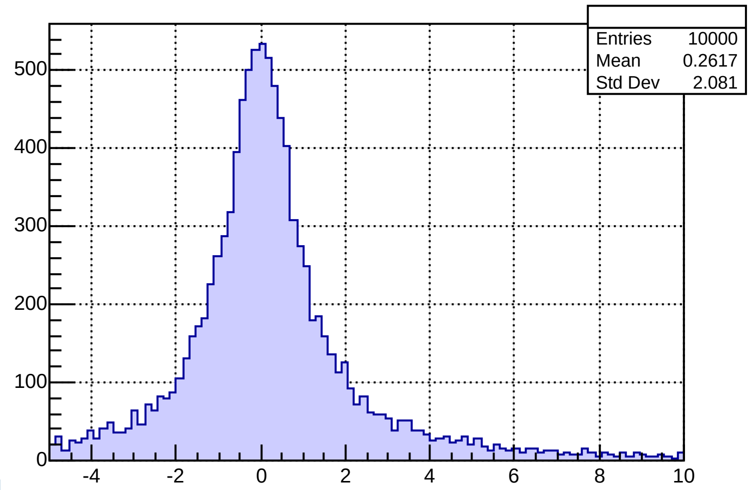

As we can see from the last figure the samples from our random number generator fit our distribution.
