# Supplementary material for: BioDynaMo: a modular platform for high-performance agent-based simulation
Source: Bioinformatics. 2021 Sep 16;38(2):453–60. doi: 10.1093/bioinformatics/btab649 (PMC8723141; doi:10.1093/bioinformatics/btab649)

Agent reproduction and mortality

Author: Lukas Breitwieser

In this tutorial we want to demonstrate how to add and remove agents from the simulation.

Let's start by setting up BioDynaMo notebooks.

In [1]:

```
%jsroot on
gROOT->LoadMacro("${BDMSYS}/etc/rootlogon.C");
```

```
INFO: Created simulation object 'simulation' with UniqueName='simulati
on'.
```

In [2]:

```
auto* ctxt = simulation.GetExecutionContext();
auto* scheduler = simulation.GetScheduler();
auto* rm = simulation.GetResourceManager();
```

Let's define our initial model: One cell at origin.

We also create an agent pointer for our cell, because raw pointers might be invalidated after a call to `Scheduler::Simulate`

In [3]:

```
auto* cell = new Cell();
ctxt->AddAgent(cell);
auto cell_aptr = cell->GetAgentPtr<Cell>();
scheduler->FinalizeInitialization();
VisualizeInNotebook();
```

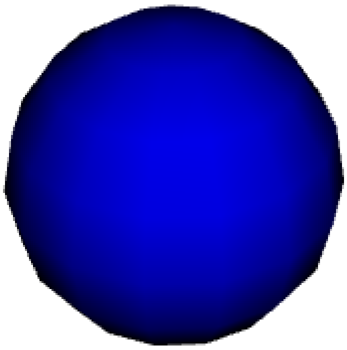

Adding an agent to the simulation is as easy as constructing one and adding it to the execution context. Our default execution context will add the new agent to the simulation at the end of the iteration. Therefore, the visualization still shows only one agent.

In [4]:

```
ctxt->AddAgent(new SphericalAgent({3, 0, 0}));
VisualizeInNotebook()
```

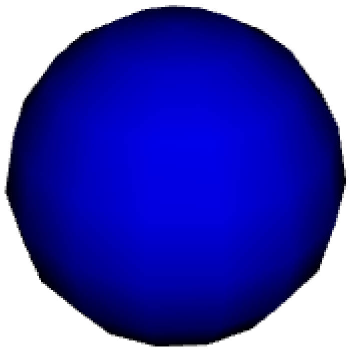

Let's simulate one time step and see what happens.

In [5]:

```
scheduler->Simulate(1);
VisualizeInNotebook()
```

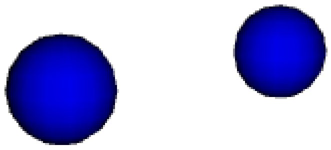

Our new agent has been added to the simulation.
Usually, new agents will be created based on some process, e.g. cell division, neurite extension from soma, neurite branching, etc. The following example shows cell division. We specify the division axis.

In [6]:

```
cell_aptr->Divide({1, 0, 0});
VisualizeInNotebook()
```

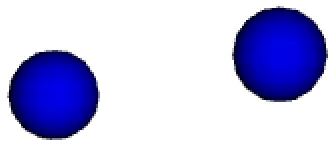

Again, the new cell is not visible yet. We have to finish one iteration.

In [7]:

```
scheduler->Simulate(1);
VisualizeInNotebook()
```

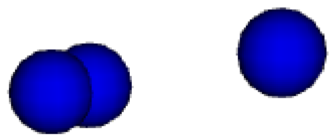

Removing agents from the simulation works similarly.
The default execution context will remove it at the end of the iteration.

In [8]:

```
cell_aptr->RemoveFromSimulation();
rm->GetNumAgents();
VisualizeInNotebook()
```

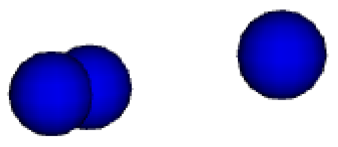

We expect that after the `Simulate` call only 2 agents are shown in the visualization.

In [9]:

```
scheduler->Simulate(1);
VisualizeInNotebook()
```

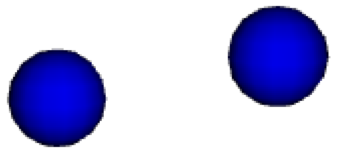

Supplement: btab649_Supplementary_Data [file btab649_supplementary_data.zip › ST03-agent-reproduction-mortality.pdf]
