# Supplementary material for: BioDynaMo: a modular platform for high-performance agent-based simulation
Source: Bioinformatics. 2021 Sep 16;38(2):453–60. doi: 10.1093/bioinformatics/btab649 (PMC8723141; doi:10.1093/bioinformatics/btab649)
Supplement: btab649_Supplementary_Data [file btab649_supplementary_data.zip › ST04-agent-reproduction-with-behaviors.pdf]

Agent reproduction with behaviors

Author: Lukas Breitwieser

In tutorial `ST3-agent-reproduction-mortality` we have explored how to add and remove agents from the simulation. In this tutorial we want to explore different behavior options to control if a new agent gets a behavior from the original agent, and if a behavior will be removed from the original one.

Let's start by setting up BioDynaMo notebooks.

In [1]:

```
%jsroot on
gROOT->LoadMacro("${BDMSYS}/etc/rootlogon.C");
```

```
INFO: Created simulation object 'simulation' with UniqueName='simulati
on'.
```

We define a simple behavior which prints `has print behavior` .

In [2]:

```
StatelessBehavior print_behavior([](Agent* agent) {
  std::cout << "  has print behavior" << std::endl;
});
```

We define the following experiment which we will run with different options of the `print_behavior` .
We create a cell, add a copy of the `print_behavior` , and run all behaviors. We expect that the following output is created.

```
mother:
  has print behavior
```

Afterwards we print a seperator `-------` to indicate cell division, divide the mother cell and run the behaviors of daughter 1 and daughter 2. By definition the original mother cell turns into daugther 1 and the new agent becomes daughter 2.

In [3]:

```
void Experiment() {
    Simulation sim("my-simulation");
    auto* mother = new Cell();
    mother->AddBehavior(print_behavior.NewCopy());
    std::cout << "mother: " << std::endl;
    mother->RunBehaviors();
    std::cout << "----------------------" << std::endl;
    auto* daughter2 = mother->Divide();
    std::cout << "mother = daughter 1: " << std::endl;
    mother->RunBehaviors(); // mother = daughter 1
    std::cout << "daughter 2: " << std::endl;
    daughter2->RunBehaviors();
}
```

Let's run the experiment with default parameters and see what happens.

In [4]:

```
Experiment();
```

```
mother:
  has print behavior
---------------------
mother = daughter 1:
  has print behavior
daughter 2:
```

The `print_behavior` was **not copied** to the daughter 2 cell and was **not removed** from the mother cell.

Let's try to copy the behavior from the mother cell to daughter 2.

In [5]:

```
print_behavior.AlwaysCopyToNew();
Experiment();
```

```
mother:
  has print behavior
---------------------
mother = daughter 1:
  has print behavior
daughter 2:
  has print behavior
```

Now the `print_behavior` **was copied** to the daughter 2 cell and was **not removed** from the mother cell.

Let's try to remove the behavior from the mother cell.

In [6]:

```
print_behavior.AlwaysCopyToNew();
print_behavior.AlwaysRemoveFromExisting();
Experiment();
```

```
mother:
  has print behavior
---------------------
mother = daughter 1:
daughter 2:
  has print behavior
```

Now the `print_behavior` **was copied** to the daughter 2 cell and **was removed** from the mother cell.

Let's reset the values to the default.

In [7]:

```
print_behavior.NeverCopyToNew();
print_behavior.NeverRemoveFromExisting();
Experiment();
```

```
mother:
  has print behavior
---------------------
mother = daughter 1:
  has print behavior
daughter 2:
```

Behaviors provide also more fine-grained distinction. Some agents support multiple new agent events (https://biodynamo.org/docs/userguide/new_agent_event/): neurite branching, neurite bifurcation, side neurite extension, etc. For each event we can specify if the behavior should be copied to the new, or removed from the existing agent.

In [8]:

```
print_behavior.CopyToNewIf({CellDivisionEvent::kUid});
print_behavior.RemoveFromExistingIf({CellDivisionEvent::kUid});
Experiment();
```

```
mother:
  has print behavior
---------------------
mother = daughter 1:
daughter 2:
  has print behavior
```
