# Supplementary material for: BioDynaMo: a modular platform for high-performance agent-based simulation
Source: Bioinformatics. 2021 Sep 16;38(2):453–60. doi: 10.1093/bioinformatics/btab649 (PMC8723141; doi:10.1093/bioinformatics/btab649)
Supplement: btab649_Supplementary_Data [file btab649_supplementary_data.zip › ST05-agent-reproduction-advanced.pdf]

Agent reproduction advanced

Author: Lukas Breitwieser

In the tutorials so far we used `Cell::Divide` to create new agents. In this demo we want to show how to define your own "process" that creates a new agent. Furthermore, we will explain the purpose of the functions `Agent::Initialize` and `Agent::Update`.

Assume that we want to create a new agent type `Human` which should be able to `GiveBirth`.

Let's start by initializing BioDynaMo notebooks.

In [1]:

```
%jsroot on
gROOT->LoadMacro("${BDMSYS}/etc/rootlogon.C");
```

```
INFO: Created simulation object 'simulation' with UniqueName='simulati
on'.
```

In [2]:

```
auto* ctxt = simulation.GetExecutionContext();
auto* scheduler = simulation.GetScheduler();
```

Let's start by creating the `ChildBirthEvent`. In this example we do not need any attributes.

In [3]:

```
struct ChildBirthEvent : public NewAgentEvent {
  ChildBirthEvent() {}
  virtual ~ChildBirthEvent() {}
  NewAgentEventUid GetUid() const override {
      static NewAgentEventUid kUid =
          NewAgentEventUidGenerator::GetInstance()->GenerateUid();
      return kUid;
  }
};
```

We continue by defining the class `Human` which derives from `SphericalAgent`.

In [4]:

```
class Human : public SphericalAgent {
  BDM_AGENT_HEADER(Human, SphericalAgent, 1);

 public:
  Human() {}
  explicit Human(const Double3& position) : Base(position) {}
  virtual ~Human() {}

  void GiveBirth();
  void Initialize(const NewAgentEvent& event) override;
};
```

The implementation of `GiveBirth` only requires two lines of code.

In [5]:

```
void Human::GiveBirth() {
    ChildBirthEvent event;
    CreateNewAgents(event, {this});
}
```

First, creating an instance of the event.
Second, invoking `CreateNewAgents` function which is defined in class `Agent`.

The first parameter of `CreateNewAgents` takes an event object, and the second a vector of agent prototypes. The size of this vector determines how many new agents will be created. In our case: one. If twins should be born we could change it to `CreateNewAgents(event, {this, this});`.

But why do we have to pass a list of agent pointers to the function?

The answer is simple: we have to tell `CreateNewAgents` which agent type it should create. In our use case we want to create another instance of class `Human`. Therefore, we pass the `this` pointer.

The only part missing is to tell BioDynaMo how to initialize the attributes of the new child. This decision is encapsulated in the `Initialize` function which we override from the base class. Don't forget to also call the implementation of the base class using `Base::Initialize(event)`. Otherwise the intialization of the base class is skipped.

In our example we define that the child should be created next to the mother in 3D space.

In [6]:

```
void Human::Initialize(const NewAgentEvent& event) {
    Base::Initialize(event);
    auto* mother = bdm_static_cast<Human*>(event.existing_agent);
    SetPosition(mother->GetPosition() + Double3{2, 0, 0});
}
```

This concludes all required building blocks. Let's try it out!

In [7]:

```
auto* human = new Human();
ctxt->AddAgent(human);
```

In [8]:

```
human->GiveBirth();
```

In [9]:

```
scheduler->Simulate(1);
VisualizeInNotebook();
```

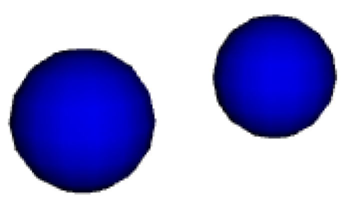

As expected the simulation consists of two "humans".

Let's take this one step further. Let's assume that class `Human` was provided in a library that we don't want to modify. However, we want to add two more attributes:

- the number of offsprings
- the mitochondiral dna (Note: the mitochondrial dna is inherited solely from the mother)

Let's create a new class called `MyHuman` which derives from `Human` and which adds these two attributes.

In [10]:

```
using MitochondrialDNA = int;
```

In [11]:

```
class MyHuman : public Human {
  BDM_AGENT_HEADER(MyHuman, Human, 1);

 public:
  MyHuman() {}
  explicit MyHuman(const Double3& position) : Base(position) {}
  virtual ~MyHuman() {}

  void Initialize(const NewAgentEvent& event) override;
  void Update(const NewAgentEvent& event) override;

  int num_offsprings_ = 0;
  MitochondrialDNA mdna_;
};
```

As in the example above, the `Initialize` method is used to set the attributes during new agent events. In this example, we have to set the mitochondrial dna of the child to the value from the mother. The following function definition does exactly that and prints out the value.

In [12]:

```
void MyHuman::Initialize(const NewAgentEvent& event) {
    Base::Initialize(event);
    auto* mother = bdm_static_cast<MyHuman*>(event.existing_agent);
    mdna_ = mother->mdna_;
    std::cout << "Initialize child attributes: mitochondrial dna set to "
              << mdna_ << std::endl;
}
```

The only task left is to update the attributes of the mother. This is done by overriding the `Update` method. Again, do not forget to call the implementation of the base class for correctness. We increment the `num_offsprings_` attribute by the number of newly created agents. Although we could just have incremented the attribute by one, the solution below is generic enough to handle e.g. twin births.

In [13]:

```
void MyHuman::Update(const NewAgentEvent& event) {
    Base::Update(event);
    num_offsprings_ += event.new_agents.size();
    std::cout << "Update mother attributes: num_offsprings incremented to "
              << num_offsprings_ << std::endl;
}
```

Let's create a new `MyHuman` , set its mitochondrial dna to `123` and output the current value of `num_offsprings_` , which we expect to be `0` .

In [14]:

```
auto* my_human = new MyHuman();
my_human->mdna_ = 123;
my_human->num_offsprings_
```

```
(int) 0
```

Now we can call `GiveBirth` again. We expect the output of two lines.

- The first coming from the child informing us about the initialization of its `mdna_` attribute
- and the second from the mother telling us about the update of `num_offsprings_`

In [15]:

```
my_human->GiveBirth();
```

```
Initialize child attributes: mitochondrial dna set to 123
Update mother attributes: num_offsprings incremented to 1
```

To double check, let's output the value of `num_offsprings` , which we expect to be 1

In [16]:

```
my_human->num_offsprings_
```

```
(int) 1
```
