# Supplementary material for: BioDynaMo: a modular platform for high-performance agent-based simulation
Source: Bioinformatics. 2021 Sep 16;38(2):453–60. doi: 10.1093/bioinformatics/btab649 (PMC8723141; doi:10.1093/bioinformatics/btab649)
Supplement: btab649_Supplementary_Data [file btab649_supplementary_data.zip › ST06-environment-search.pdf]

Environment search

Author: Lukas Breitwieser

In this tutorial we will show how to execute a function for each neighbor of an agent.

Let's start by setting up BioDynaMo notebooks.

In [1]:

```
%jsroot on
gROOT->LoadMacro("${BDMSYS}/etc/rootlogon.C");
```

```
INFO: Created simulation object 'simulation' with UniqueName='simulati
on'.
```

We create three agents in a row along the x-axis with identical y and z values.

In [2]:

```
auto* ctxt = simulation.GetExecutionContext();

auto* a0 = new SphericalAgent({10, 0, 0});
auto* a1 = new SphericalAgent({20, 0, 0});
auto* a2 = new SphericalAgent({30, 0, 0});

a0->SetDiameter(11);
a1->SetDiameter(11);
a2->SetDiameter(11);

ctxt->AddAgent(a0);
ctxt->AddAgent(a1);
ctxt->AddAgent(a2);
```

We finalize the initialization and update the environment so it can be used later. Please not that this is usually done automatically inside `Scheduler::Simulate` .

In [3]:

```
simulation.GetScheduler()->FinalizeInitialization();
simulation.GetEnvironment()->Update();
VisualizeInNotebook();
```

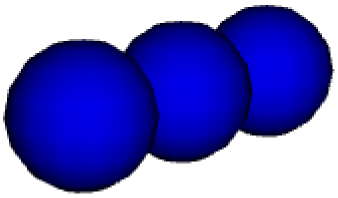

Let's define the function that we want to execute for each neighbor. It prints the unique id of the neighbor and its distance from the querying agent.

In [4]:

```
auto print_id_distance = L2F([](Agent* a, double squared_distance) {
  std::cout << "Neighbor " << a->GetUid() << "  with distance: "
            << std::sqrt(squared_distance) << std::endl;
});
```

The agents have the following ids (in order of increasing x-value) 0-0, 1-0, 2-0

We start by executing print_id_distance for the first agent. We ask for all neighbors within distance 101. Therefore the function should be executed for the agent in the middle with id 1-0

In [5]:

```
ctxt->ForEachNeighbor(print_id_distance, *a0, 101);
```

```
Neighbor 1-0  with distance: 10
```

Let's repeat the experiment for the middle agent. We expect to see two lines for the left and right neighbor.

In [6]:

```
ctxt->ForEachNeighbor(print_id_distance, *a1, 101);
```

```
Neighbor 0-0  with distance: 10
Neighbor 2-0  with distance: 10
```

Lastly, we want to execute the function `print_id_distance` for all neighbors of the righ-most agent. We expect to see one line printing the middle agent as neighbor (1-0)

In [7]:

```
ctxt->ForEachNeighbor(print_id_distance, *a2, 101);
```

```
Neighbor 1-0  with distance: 10
```
