# Supplementary material for: BioDynaMo: a modular platform for high-performance agent-based simulation
Source: Bioinformatics. 2021 Sep 16;38(2):453–60. doi: 10.1093/bioinformatics/btab649 (PMC8723141; doi:10.1093/bioinformatics/btab649)
Supplement: btab649_Supplementary_Data [file btab649_supplementary_data.zip › ST07-multi-scale-simulation.pdf]

Multi-scale simulations

Author: Lukas Breitwieser

In this tutorial we will show how BioDynaMo support multi-scale simulations. Multi-scale simulation means that simulated processes happen in different time-scales---e.g. substance diffusion and neurite growth.

Let's start by setting up BioDynaMo notebooks.

In [1]:

```
%jsroot on
gROOT->LoadMacro("${BDMSYS}/etc/rootlogon.C");
```

```
INFO: Created simulation object 'simulation' with UniqueName='simulati
on'.
```

We define a new [standalone operation (https://biodynamo.org/docs/userguide/operation/)](https://biodynamo.org/docs/userguide/operation/) which only task is to print the current simulation time step if it is executed.

In [2]:

```
struct TestOp : public StandaloneOperationImpl {
  BDM_OP_HEADER(TestOp);
  void operator()() override {
    auto* scheduler = Simulation::GetActive()->GetScheduler();
    auto* param = Simulation::GetActive()->GetParam();
    std::cout << "Processing iteration "
              << scheduler->GetSimulatedSteps()
              << " simulation time "
              << scheduler->GetSimulatedSteps() * param->simulation_time_step
              << std::endl;
  }
};
OperationRegistry::GetInstance()->AddOperationImpl(
    "test_op", OpComputeTarget::kCpu, new TestOp());
```

In [3]:

```
auto set_param = [](Param * param) {
    param->simulation_time_step = 2;
};
Simulation simulation("my-simulation", set_param);
```

Our initial model consists of one agent at origin.

In [4]:

```
auto* ctxt = simulation.GetExecutionContext();
ctxt->AddAgent(new SphericalAgent());
```

Let's create a new instance of our class `TestOp` and add it to the scheduler.

In [5]:

```
auto* op1 = NewOperation("test_op");
auto* scheduler = simulation.GetScheduler();
scheduler->ScheduleOp(op1);
```

Let's simulate 9 steps. We expect that `op1` will be called each time step.

In [6]:

```
scheduler->Simulate(9);
```

```
Processing iteration 0 simulation time 0
Processing iteration 1 simulation time 2
Processing iteration 2 simulation time 4
Processing iteration 3 simulation time 6
Processing iteration 4 simulation time 8
Processing iteration 5 simulation time 10
Processing iteration 6 simulation time 12
Processing iteration 7 simulation time 14
Processing iteration 8 simulation time 16
```

Operations have a frequency attribute which specifies how often it will be executed. An operation with frequency one will be executed at every time step; an operation with frequency two every second, and so on.

In [7]:

```
op1->frequency_= 3;
scheduler->Simulate(9);
```

```
Processing iteration 9 simulation time 18
Processing iteration 12 simulation time 24
Processing iteration 15 simulation time 30
```

This functionality can be used to set the frequency of different processes in an agent-based model.
