# Supplementary material for: BioDynaMo: a modular platform for high-performance agent-based simulation
Source: Bioinformatics. 2021 Sep 16;38(2):453–60. doi: 10.1093/bioinformatics/btab649 (PMC8723141; doi:10.1093/bioinformatics/btab649)
Supplement: btab649_Supplementary_Data [file btab649_supplementary_data.zip › ST08-histograms.pdf]

Create a histogram of agent attributes

Author: Lukas Breitwieser

In this tutorial we will show how to create a histogram of all agent diameters in the simulation and fit a function to the data.

Let's start by setting up BioDynaMo notebooks.

In [1]:

```
%jsroot on
gROOT->LoadMacro("${BDMSYS}/etc/rootlogon.C");
```

```
INFO: Created simulation object 'simulation' with UniqueName='simulati
on'.
```

We want to define a function that creates a cell at a certain position with diameters drawn from a gaussian distribution with $\mu = 20$ and $\sigma = 5$. The smallest diameter should be larger then 2.0.

In [2]:

```
simulation.GetResourceManager()->ClearAgents();
auto rng = simulation.GetRandom()->GetGausRng(20, 5);
auto create_cell = [&](const Double3& position) {
  Cell* cell = new Cell(position);
  double diameter = std::max(2.0, rng.Sample());
  cell->SetDiameter(diameter);
  return cell;
};
```

Now that we defined `create_cell` we can use it to create 400 cells on a plane with $z = 0$, $xmin = ymin = -200$, $xmax = ymax = 200$, and spacing = 20 in both dimensions.

In [3]:

```
auto f = [](const double* x, const double* params) { return 0.0; };
ModelInitializer::CreateAgentsOnSurface(f, {}, -200, 200, 20, -200, 200, 20,
                                        create_cell);
simulation.GetScheduler()->FinalizeInitialization();
VisualizeInNotebook(300, 300);
```

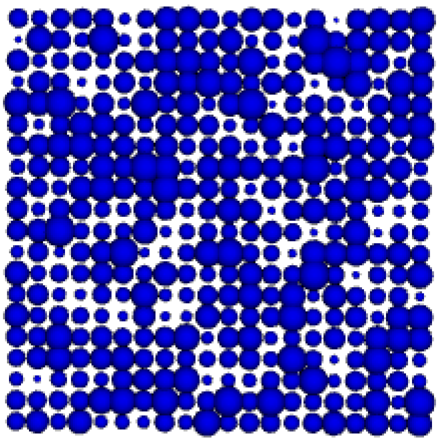

The next step is to create a histogram object with 100 bins in the interval [2, 40].
The second line creates a function which fills the histogram with the diameter of the given agent.
The third line calls the function `fill` for each agent, thus adding all diameters to the histogram.

In [4]:

```
TH1F h("myHisto","Agent Diameter Histogram;Diameter;Count", 100, 2, 40);
auto fill = L2F([&](Agent* a, AgentHandle){ h.Fill(a->GetDiameter()); });
simulation.GetResourceManager()->ForEachAgent(fill);
```

Let's draw the final histogram.
Before we have to create a `TCanvas` object in order to display the result in this notebook.
We also modify the default color and create a grid.

In [5]:

```
TCanvas c("", "", 400, 300);
h.SetFillColor(kBlue - 10);
c.SetGrid();
h.Draw();
c.Draw();
```

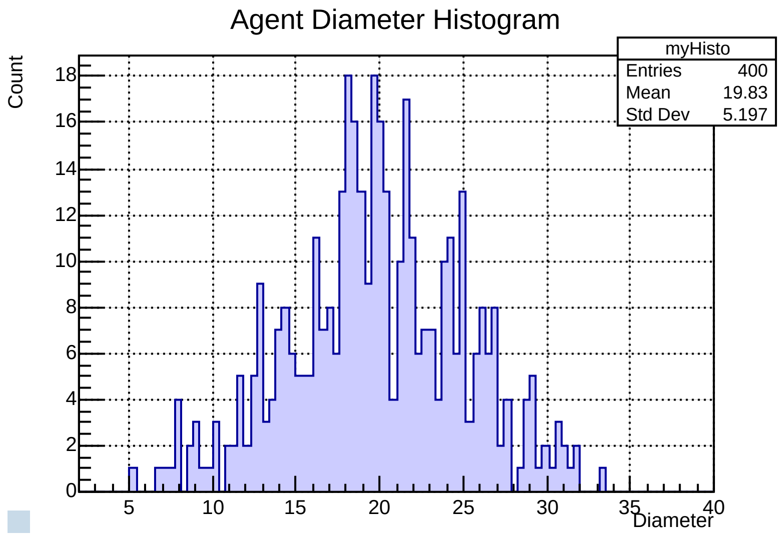

Finally, we can try to fit a function to the data in the histogram.
Since we drew samples from a gaussian random number generator when we created our cells, we expect that a gaussian will fit our data.

In [6]:

```
h.Fit("gaus", "S");
h.Draw();
c.Draw();
```

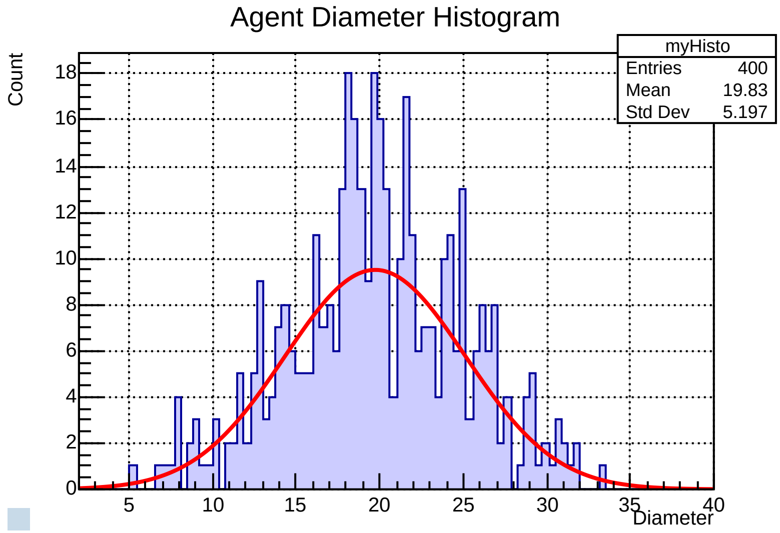


```
 FCN=73.1872 FROM MIGRAD    STATUS=CONVERGED      78 CALLS          79
TOTAL
                     EDM=6.76682e-08    STRATEGY= 1      ERROR MATRIX
ACCURATE
  EXT PARAMETER                                   STEP         FIRST
  NO.   NAME      VALUE            ERROR          SIZE      DERIVATIVE
   1  Constant     9.50544e+00   7.09907e-01   2.21048e-03   4.02840e-
04
   2  Mean         1.97314e+01   3.23457e-01   1.36014e-03  -3.65236e-
04
   3  Sigma        5.40166e+00   3.17423e-01   6.23484e-05   2.34242e-
02
```
