# Supplementary material for: BioDynaMo: a modular platform for high-performance agent-based simulation
Source: Bioinformatics. 2021 Sep 16;38(2):453–60. doi: 10.1093/bioinformatics/btab649 (PMC8723141; doi:10.1093/bioinformatics/btab649)

Simulation time series plotting (basics)

Author: Lukas Breitwieser

In this tutorial we show how to collect data during the simulation and plot it at the end.
To this extent, we create a simulation where cells divide rapidly leading to exponential growth.

Let's start by setting up BioDynaMo notebooks.

In [1]:

```
%jsroot on
gROOT->LoadMacro("${BDMSYS}/etc/rootlogon.C");
```

```
INFO: Created simulation object 'simulation' with UniqueName='simulati
on'.
```

In [2]:

```
using namespace bdm::experimental;
```

In [3]:

```
auto set_param = [](Param* param) {
    param->simulation_time_step = 1.0;
};
Simulation simulation("MySimulation", set_param);
```

Let's create a behavior which divides cells with 10% probability in each time step.
New cells should also get this behavior.
Therefore, we have to call `AlwaysCopyToNew()` .
Otherwise, we would only see linear growth.

In [4]:

```
StatelessBehavior rapid_division([](Agent* agent) {
  if (Simulation::GetActive()->GetRandom()->Uniform() < 0.1) {
    bdm_static_cast<Cell*>(agent)->Divide();
  }
});
rapid_division.AlwaysCopyToNew();
```

Let's create a function that creates a cell at a specific position, with diameter = 10, and the `rapid_division` behavior.

In [5]:

```
auto create_cell = [](const Double3& position) {
  Cell* cell = new Cell(position);
  cell->SetDiameter(10);
  cell->AddBehavior(rapid_division.NewCopy());
  return cell;
};
```

As starting condition we want to create 100 cells randomly distributed in a cube with $min = 0, max = 200$

In [6]:

```
simulation.GetResourceManager()->ClearAgents();
ModelInitializer::CreateAgentsRandom(0, 200, 100, create_cell);
simulation.GetScheduler()->FinalizeInitialization();
VisualizeInNotebook();
```

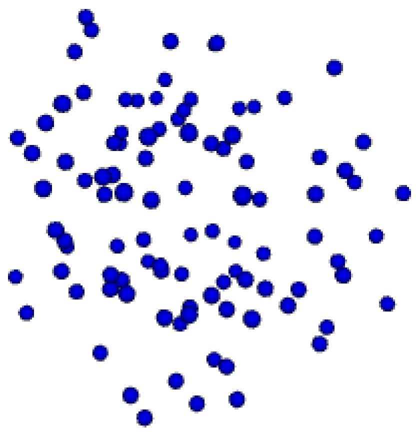

Before we start the simulation, we have to tell BioDynaMo which data to collect.
We can do this with the `TimeSeries::AddCollector` function. In this example we are interested in the number of agents.

In [7]:

```
auto* ts = simulation.GetTimeSeries();
auto get_num_agents = [](Simulation* sim) {
  return static_cast<double>(sim->GetResourceManager()->GetNumAgents());
};
ts->AddCollector("num-agents", get_num_agents);
```

Now let's simulate until there are 4000 agents in the simulation

In [8]:

```
auto exit_condition = [](){
    auto* rm = Simulation::GetActive()->GetResourceManager();
    return rm->GetNumAgents() > 4000;
};
simulation.GetScheduler()->SimulateUntil(exit_condition);
```

Now we can plot how the number of agents (in this case cells) evolved over time.

In [9]:

```
LineGraph g(ts, "My result", "Time", "Number of agents", true, nullptr, 500, 300);
g.Add("num-agents", "Number of Agents");
g.Draw();
```

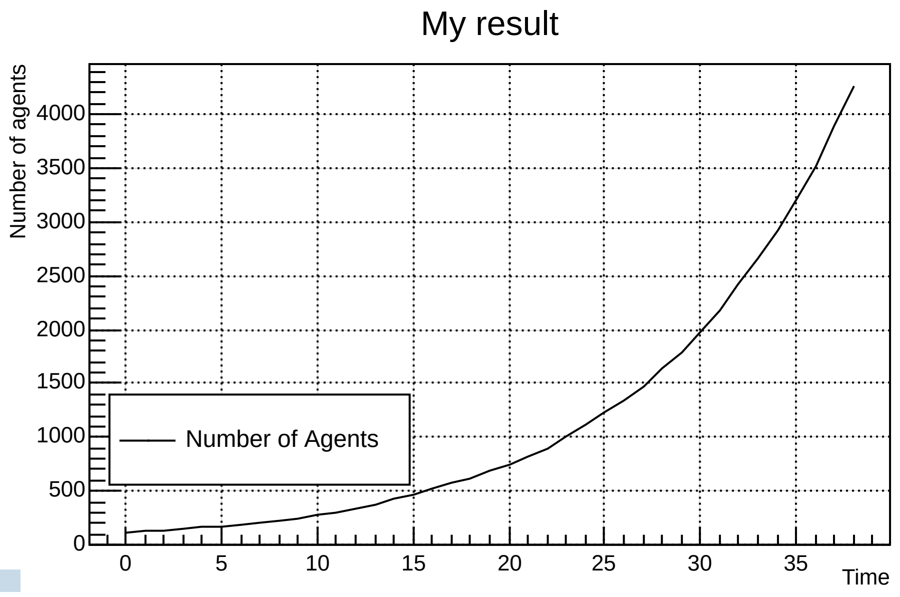

Supplement: btab649_Supplementary_Data [file btab649_supplementary_data.zip › ST09-timeseries-plotting-basic.pdf]
