# Supplementary material for: BioDynaMo: a modular platform for high-performance agent-based simulation
Source: Bioinformatics. 2021 Sep 16;38(2):453–60. doi: 10.1093/bioinformatics/btab649 (PMC8723141; doi:10.1093/bioinformatics/btab649)
Supplement: btab649_Supplementary_Data [file btab649_supplementary_data.zip › ST10-timeseries-plotting-and-analysis.pdf]

Simulation time series plotting and analysis

Author: Lukas Breitwieser

In this tutorial we show how to collect data during the simulation, and plot and analyse it at the end.
To this extent, we create a simulation where cells divide rapidly leading to exponential growth.

Let's start by setting up BioDynaMo notebooks.

In [1]:

```
%jsroot on
gROOT->LoadMacro("${BDMSYS}/etc/rootlogon.C");
```

```
INFO: Created simulation object 'simulation' with UniqueName='simulati
on'.
```

In [2]:

```
using namespace bdm::experimental;
```

In [3]:

```
auto set_param = [](Param* param) {
    param->simulation_time_step = 1.0;
};
Simulation simulation("MySimulation", set_param);
```

Let's create a behavior which divides cells with 5% probability in each time step.
New cells should also get this behavior.
Therefore, we have to call `AlwaysCopyToNew()` .
Otherwise, we would only see linear growth.

In [4]:

```
StatelessBehavior rapid_division([](Agent* agent) {
  if (Simulation::GetActive()->GetRandom()->Uniform() < 0.05) {
    bdm_static_cast<Cell*>(agent)->Divide();
  }
});
rapid_division.AlwaysCopyToNew();
```

Let's create a function that creates a cell at a specific position, with diameter = 10, and the `rapid_division` behavior.

In [5]:

```
auto create_cell = [](const Double3& position) {
  Cell* cell = new Cell(position);
  cell->SetDiameter(10);
  cell->AddBehavior(rapid_division.NewCopy());
  return cell;
};
```

As starting condition we want to create 100 cells randomly distributed in a cube with $min = 0, max = 200$

In [6]:

```
simulation.GetResourceManager()->ClearAgents();
ModelInitializer::CreateAgentsRandom(0, 200, 100, create_cell);
simulation.GetScheduler()->FinalizeInitialization();
VisualizeInNotebook();
```

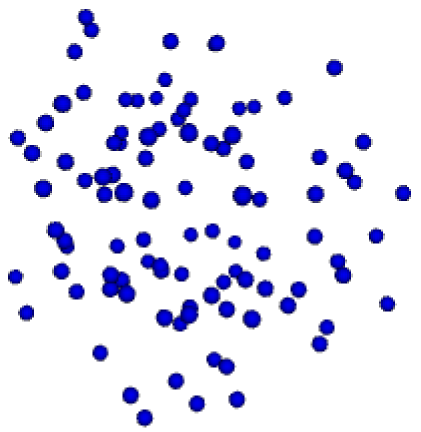

Before we start the simulation, we have to tell BioDynaMo which data to collect.
We can do this with the TimeSeries::AddCollector function.
In this example we are interested in the number of agents ...

In [7]:

```
auto* ts = simulation.GetTimeSeries();
auto get_num_agents = [](Simulation* sim) {
  return static_cast<double>(sim->GetResourceManager()->GetNumAgents());
};
ts->AddCollector("num-agents", get_num_agents);
```

... and the number agents with $diameter < 5$.
We create a condition `cond` and pass it to the function `Count` which returns the number of agents for which `cond(agent)` evaluates to true.

In [8]:

```
auto* ts = simulation.GetTimeSeries();
auto agents_lt_5 = [](Simulation* sim) {
  auto cond = L2F([](Agent* a){ return a->GetDiameter() < 5; });
  return static_cast<double>(bdm::experimental::Count(sim, cond));
};
ts->AddCollector("agents_lt_5", agents_lt_5);
```

Now let's simulate 40 iterations

In [9]:

```
simulation.GetScheduler()->Simulate(40);
```

Now we can plot how the number of agents (in this case cells) and the number of agents with $diameter < 5$ evolved over time.

In [10]:

```
LineGraph g(ts, "my result", "Time", "Number of agents",
                               true, nullptr, 500, 300);
g.Add("num-agents", "Number of Agents", "L", kBlue);
g.Add("agents_lt_5", "Number of Agents diam < 5", "L", kGreen);
g.Draw();
```

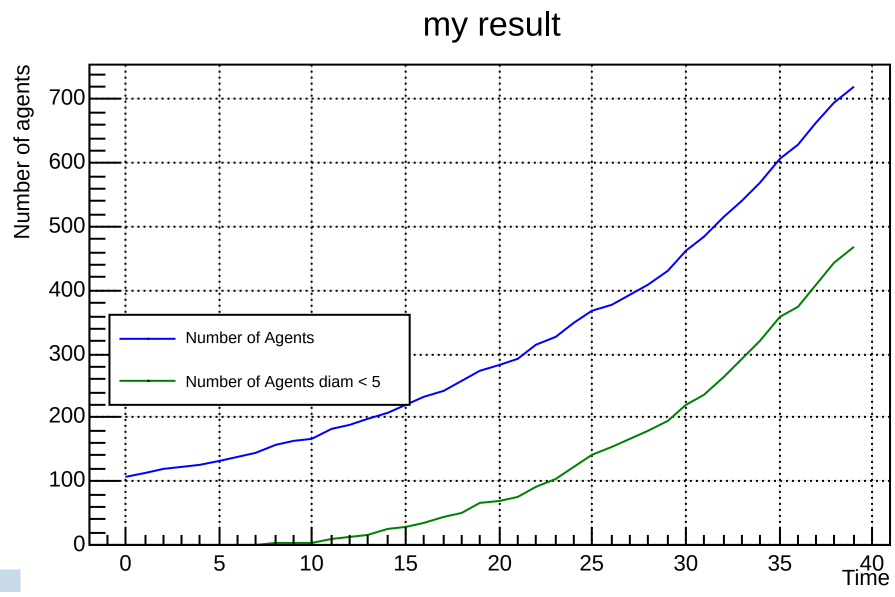

Let's try to fit an exponential function to verify our assumption that the cells grew exponentially.
Please visit the [ROOT user-guide (https://root.cern.ch/root/htmldoc/guides/users-guide/FittingHistograms.html)](https://root.cern.ch/root/htmldoc/guides/users-guide/FittingHistograms.html) for more information regarding fitting

In [11]:

```
auto fitresult = g.GetTGraphs("num-agents")[0]->Fit("expo", "S");
g.Draw();
```

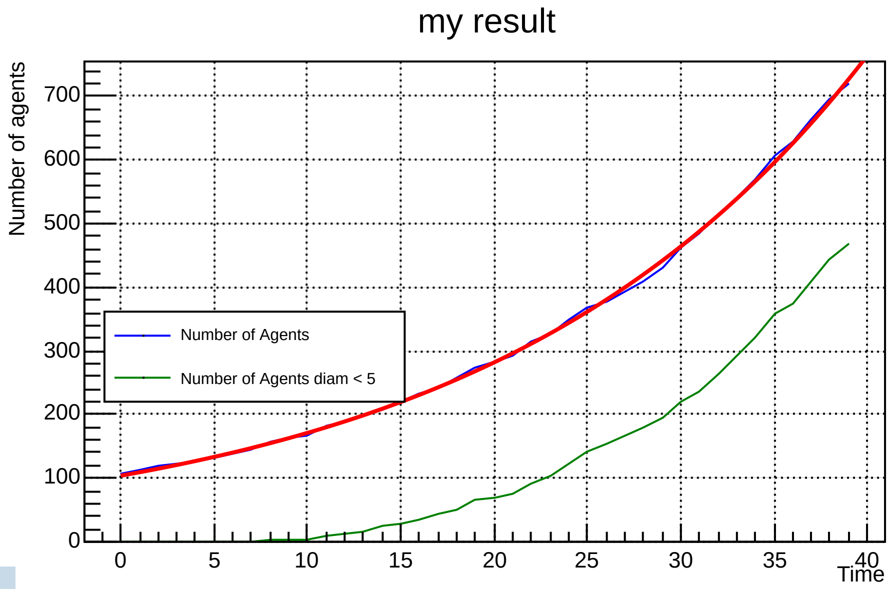


```
****************************************
Minimizer is Minuit / Migrad
Chi2                      =      793.088
NDf                       =           38
Edm                       =  1.27707e-08
NCalls                    =           49
Constant                  =      4.64437   +/-   0.00734877
Slope                     =    0.0498693   +/-   0.000234423
```

Indeed, the number of agents follow an exponential function

$$y = \exp(slope * x + constant)$$

with constant = 4.6 and slope = 0.049 This corresponds to the division probability of 0.05

This is how to change the color after the creation of `g`.
Also the position of the legend can be optimized.

In [12]:

```
g.GetTGraphs("num-agents")[0]->SetLineColor(kBlack);
g.SetLegendPos(1, 500, 20, 700);
g.Draw();
```

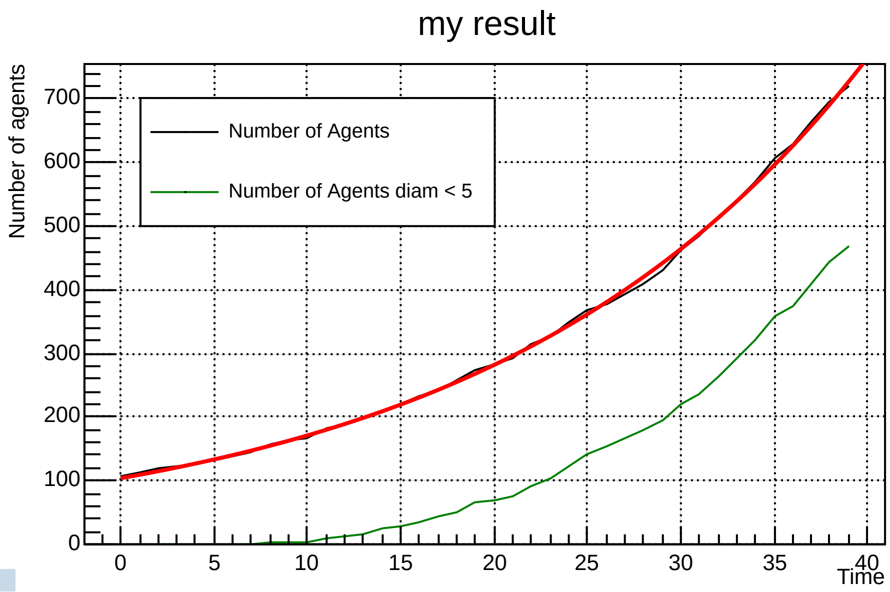

Let's save these results in multiple formats

In [13]:

```
g.SaveAs(Concat(simulation.GetOutputDir(), "/line-graph"),
         {".root", ".svg", ".png", ".C"});
```
