# Supplementary material for: BioDynaMo: a modular platform for high-performance agent-based simulation
Source: Bioinformatics. 2021 Sep 16;38(2):453–60. doi: 10.1093/bioinformatics/btab649 (PMC8723141; doi:10.1093/bioinformatics/btab649)
Supplement: btab649_Supplementary_Data [file btab649_supplementary_data.zip › ST11-multiple-experiments-statistical-analysis.pdf]

Multiple experiments and statistical analysis

Author: Lukas Breitwieser
In this tutorial we show how to collect and analyse data from multiple experiments.
To this extent, we create a simulation where cells divide rapidly leading to exponential growth.

Let's start by setting up BioDynaMo notebooks.

In [1]:

```
%jsroot on
gROOT->LoadMacro("${BDMSYS}/etc/rootlogon.C");
```

```
INFO: Created simulation object 'simulation' with UniqueName='simulati
on'.
```

In [2]:

```
using namespace bdm::experimental;
```

In [3]:

```
double gDivProb = 0.05;
```

We use the same simulation as in `ST09-timeseries-plotting-basic` . It is a simulation were agents divide with a specific division probability in each time step leading to exponential growth. We collect the number of agents in each time step. Have a look at `ST09-timeseries-plotting-basic` for more information.

We wrap the required simulation code in a function called `Experiment` which takes two parameters:

- the collected result data from a single invocation (output param)
- the division probability parameter

In [4]:

```
void Experiment(TimeSeries* result, double division_probability) {
    gDivProb = division_probability;

    auto set_param = [](Param* param) {
        param->simulation_time_step = 1.0;
    };
    Simulation simulation("MySimulation", set_param);

    StatelessBehavior rapid_division([](Agent* agent) {
      if (Simulation::GetActive()->GetRandom()->Uniform() < gDivProb) {
        bdm_static_cast<Cell*>(agent)->Divide();
      }
    });
    rapid_division.AlwaysCopyToNew();

    auto create_cell = [&](const Double3& position) {
      Cell* cell = new Cell(position);
      cell->SetDiameter(10);
      cell->AddBehavior(rapid_division.NewCopy());
      return cell;
    };

    simulation.GetResourceManager()->ClearAgents();
    ModelInitializer::CreateAgentsRandom(0, 200, 100, create_cell);
    simulation.GetScheduler()->FinalizeInitialization();

    auto* ts = simulation.GetTimeSeries();
    auto get_num_agents = [](Simulation* sim) {
      return static_cast<double>(sim->GetResourceManager()->GetNumAgents());
    };
    ts->AddCollector("num-agents", get_num_agents);

    simulation.GetScheduler()->Simulate(40);

    // move collected time series data from simulation to object result
    *result = std::move(*simulation.GetTimeSeries());
}
```

We want to run our experiment for 10 times with a different division probability parameter. We choose the division probability randomly between 0.04 and 0.06

In [5]:

```
std::vector<TimeSeries> individual_results(10);
Random rnd;
for(auto& ir : individual_results) {
    Experiment(&ir, rnd.Uniform(0.04, 0.06));
}
```

In the next step we want to combine the individual results. Therefore we calculate the mean, and min (error low), and max (error high) and store it in a merged `TimeSeries` object.

In [6]:

```
TimeSeries merged;
auto merger = [](const std::vector<double>& all_ys,
                  double* y, double* eh, double* el) {
    *y = TMath::Mean(all_ys.begin(), all_ys.end());
    *el = *y - *TMath::LocMin(all_ys.begin(), all_ys.end());
    *eh = *TMath::LocMax(all_ys.begin(), all_ys.end()) - *y;
};
TimeSeries::Merge(&merged, individual_results, merger);
```

Now we can print the merged results and see how the simulations evolved over time, and how they differed from each other.

In [7]:

```
LineGraph g(&merged, "My result", "Time", "Number of agents", false, nullptr, 500,
g.Add("num-agents", "Number of Agents", "LP", kBlue);
g.Draw();
```

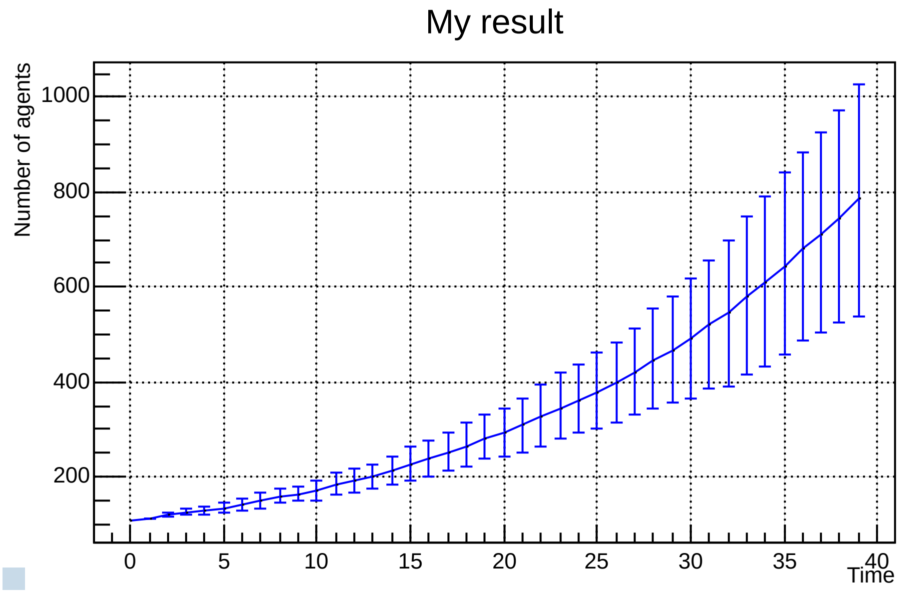

Finally, let's fit an exponential function to the data.

In [8]:

```
g.GetTMultiGraph()->Fit("expo", "S");
g.Draw()
```

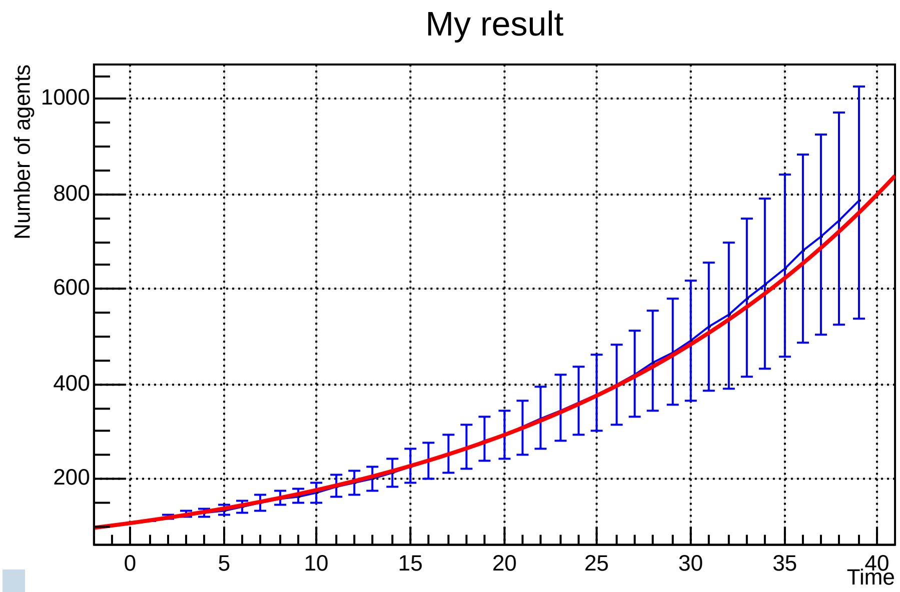


```
 FCN=0.850945 FROM MIGRAD    STATUS=CONVERGED      39 CALLS          4
0 TOTAL
                     EDM=6.73798e-11    STRATEGY= 1      ERROR MATRIX
ACCURATE
  EXT PARAMETER                                   STEP         FIRST
  NO.   NAME      VALUE            ERROR          SIZE      DERIVATIVE
   1  Constant     4.67133e+00   1.58086e-02   8.77643e-06   8.20004e-
04
   2  Slope        5.03276e-02   1.84650e-03   1.02514e-06   6.11443e-
03
```
