# Supplementary material for: BioDynaMo: a modular platform for high-performance agent-based simulation
Source: Bioinformatics. 2021 Sep 16;38(2):453–60. doi: 10.1093/bioinformatics/btab649 (PMC8723141; doi:10.1093/bioinformatics/btab649)
Supplement: btab649_Supplementary_Data [file btab649_supplementary_data.zip › ST12-hierarchical-model.pdf]

Hierarchical model support

Author: Lukas Breitwieser

Some models require to update certain agents before others. In this tutorial we show how to execute operations first for large agents and afterwards for small ones. Lastly, we demonstrate how to run a different set of operations for large and for small agents.

Let's start by setting up BioDynaMo notebooks.

In [1]:

```
%jsroot on
gROOT->LoadMacro("${BDMSYS}/etc/rootlogon.C");
```

```
INFO: Created simulation object 'simulation' with UniqueName='simulati
on'.
```

To make this demo easier to understand, we turn off multi-threading and load balancing.

In [2]:

```
omp_set_num_threads(1);
ThreadInfo::GetInstance()->Renew();
auto* scheduler = simulation.GetScheduler();
scheduler->UnscheduleOp(scheduler->GetOps("load balancing")[0]);
```

We create a new agent operation which prints out its name and the diameter of the agent it is processing

In [3]:

```
struct TestOp : public AgentOperationImpl {
  BDM_OP_HEADER(TestOp);
  void operator()(Agent* agent) override {
    std::cout << name << " processing agent with diameter "
              << agent->GetDiameter() << endl;
  }
  std::string name = "";
};
OperationRegistry::GetInstance()->AddOperationImpl(
    "test_op", OpComputeTarget::kCpu, new TestOp());
```

We create four agents with diameter `{20, 10, 20, 10}`

In [4]:

```
auto* ctxt = simulation.GetExecutionContext();
for (int i = 0; i < 4; ++i) {
    double diameter = i % 2 == 0 ? 20 : 10;
    ctxt->AddAgent(new SphericalAgent(diameter));
}
```

We add the new operation to the simulation

In [5]:

```
auto* op1 = NewOperation("test_op");
scheduler->ScheduleOp(op1);
```

Let's simulate one time step and observe the default behavior of BioDynaMo. We expect that the agents are processed in the order they were added (`{20, 10, 20, 10}`)

In [6]:

```
scheduler->Simulate(1);
```

```
processing agent with diameter 20
processing agent with diameter 10
processing agent with diameter 20
processing agent with diameter 10
```

Now we want to define the group of large and small agents and tell BioDynaMo that large agents should be processed before small ones.

This can be done with the following three lines of code.

In [7]:

```
auto small_filter = L2F([](Agent* a) { return a->GetDiameter() < 15; });
auto large_filter = L2F([](Agent* a) { return a->GetDiameter() >= 15; });
scheduler->SetAgentFilters({&large_filter, &small_filter});
```

Let's observe if the output has changed. We expect to see first the large agents `{20, 20}` , followed by the small ones `{10, 10}` .

In [8]:

```
scheduler->Simulate(1);
```

```
processing agent with diameter 20
processing agent with diameter 20
processing agent with diameter 10
processing agent with diameter 10
```

Let's create two more instances of our `TestOp` . We define that:

- `op1` should be run for all agents (large and small).
- `op2` only for small agents
- `op3` only for large agents

In [9]:

```
auto* op2 = NewOperation("test_op");
auto* op3 = NewOperation("test_op");

op1->GetImplementation<TestOp>()->name = "OpAll      ";
op2->GetImplementation<TestOp>()->name = "OpOnlySmall";
op3->GetImplementation<TestOp>()->name = "OpOnlyLarge";

op2->SetExcludeFilters({&large_filter});
op3->SetExcludeFilters({&small_filter});

scheduler->ScheduleOp(op2);
scheduler->ScheduleOp(op3);
```

Now we want to execute another time step with the updated model. We expect that for each agent two operations will be executed.

For large agents `OpAll` and `OpOnlyLarge` and for small agents `OpAll` and `OpOnlySmall` . As before, we expect that first all large agents are executed, followed by all small agents.

In [10]:

```
scheduler->Simulate(1);
```

```
OpAll       processing agent with diameter 20
OpOnlyLarge processing agent with diameter 20
OpAll       processing agent with diameter 20
OpOnlyLarge processing agent with diameter 20
OpAll       processing agent with diameter 10
OpOnlySmall processing agent with diameter 10
OpAll       processing agent with diameter 10
OpOnlySmall processing agent with diameter 10
```
