# Supplementary material for: BioDynaMo: a modular platform for high-performance agent-based simulation
Source: Bioinformatics. 2021 Sep 16;38(2):453–60. doi: 10.1093/bioinformatics/btab649 (PMC8723141; doi:10.1093/bioinformatics/btab649)
Supplement: btab649_Supplementary_Data [file btab649_supplementary_data.zip › ST13-dynamic-scheduling.pdf]

Dynamic scheduling

Author: Lukas Breitwieser
This tutorial demonstrates that behaviors and operations can be added and removed during the simulation. This feature provides maximum flexibility to control which functions will be executed during the lifetime of a simulation.

Let's start by setting up BioDynaMo notebooks.

In [1]:

```
%jsroot on
gROOT->LoadMacro("${BDMSYS}/etc/rootlogon.C");
```

```
INFO: Created simulation object 'simulation' with UniqueName='simulati
on'.
```

In [2]:

```
auto* ctxt = simulation.GetExecutionContext();
auto* scheduler = simulation.GetScheduler();
```

Define a helper variable

In [3]:

```
int test_op_id = 0;
```

We define a standalone operation `TestOp` which prints out that it got executed and which removes itself from the list of scheduled operations afterwards. The same principles apply also for agent operations.

In [4]:

```
struct TestOp : public StandaloneOperationImpl {
  BDM_OP_HEADER(TestOp);
  void operator()() override {
    auto* scheduler = Simulation::GetActive()->GetScheduler();
    std::cout << name << " processing iteration "
              << scheduler->GetSimulatedSteps()
              << std::endl;

    auto* op = scheduler->GetOps("test_op")[test_op_id++];
    scheduler->UnscheduleOp(op);

    std::cout << "   " << name
          << " removed itself from the simulation " << std::endl;
  }
  std::string name = "";
};
OperationRegistry::GetInstance()->AddOperationImpl(
    "test_op", OpComputeTarget::kCpu, new TestOp());
```

Let's define a little helper function which creates a new instance of `TestOp` and adds it to the list of scheduled operations.

In [5]:

```
void AddNewTestOpToSim(const std::string& name) {
    auto* op = NewOperation("test_op");
    op->GetImplementation<TestOp>()->name = name;
    scheduler->ScheduleOp(op);
}
```

Let's define a new behavior `b2` which prints out when it gets executed and which adds a new operation with name `OP2` to the simulation if a condition is met.

In this scenario the condition is defined as `simulation time step == 1`.

In [6]:

```
StatelessBehavior b2([](Agent* agent) {
    std::cout << "B2 " << agent->GetUid() << std::endl;
    if (simulation.GetScheduler()->GetSimulatedSteps() == 1) {
        AddNewTestOpToSim("OP2");
        std::cout << "  B2 added OP2 to the simulation" << std::endl;
    }
});
```

We define another behavior `b1` which prints out when it gets executed, removes itself from the agent, and which adds behavior `b2` to the agent.

In [7]:

```
StatelessBehavior b1([](Agent* agent) {
    std::cout << "B1 " << agent->GetUid() << std::endl;
    agent->RemoveBehavior(agent->GetAllBehaviors()[0]);
    std::cout << "  B1 removed itself from agent " << agent->GetUid() << std::endl;
    agent->AddBehavior(b2.NewCopy());
    std::cout << "  B1 added B2 to agent " << agent->GetUid() << std::endl;
});
```

Now all required building blocks are ready. Let's define the initial model: a single agent with behavior `b1`.

In [8]:

```
auto* agent = new SphericalAgent();
agent->AddBehavior(b1.NewCopy());
ctxt->AddAgent(agent);
```

We also add a new operation to the simulation.

In [9]:

```
AddNewTestOpToSim("OP1");
```

Let's simulate one iteration and think about the expected output.

- Since we initialized our only agent with behavior `b1`, we expect to see a line `B1 0-0`
- Furthermore, `b1` will print a line to inform us that it removed itself from the agent, and that it added behavior `b2` to the agent.

- Because changes are applied immediately (using the default `InPlaceExecCtxt`) also `B2` will be executed. However the condition inside `b2` is not met.
- Next we expect an output from `OP1` telling us that it got executed.
- Lastly, we expect an output from `OP1` to tell is that it removed itself from the simulation.

In [10]:

```
scheduler->Simulate(1);
```

```
B1 0-0
  B1 removed itself from agent 0-0
  B1 added B2 to agent 0-0
B2 0-0
OP1 processing iteration 0
  OP1 removed itself from the simulation
```

Let's simulate another iteration.
This time we only expect output from `B2` . Remember that `B1` and `OP1` have been removed in the last iteration.

This time the condition in `B2` is met and we expect to see an output line to tell us that a new instance of `TestOp` with name `OP2` has been added to the simulation.

In [11]:

```
scheduler->Simulate(1);
```

```
B2 0-0
  B2 added OP2 to the simulation
```

Let's simulate another iteration. This time we expect an output from `B2` whose condition is not met in this iterations, and from `OP2` that it got executed and removed from the simulation.

In [12]:

```
scheduler->Simulate(1);
```

```
B2 0-0
OP2 processing iteration 2
  OP2 removed itself from the simulation
```

Let's simulate one last iteration. `OP2` removed itself in the last iteration. Therefore, only `B2` should be left. The condition of `B2` is not met.

In [13]:

```
scheduler->Simulate(1);
```

```
B2 0-0
```

In summary: We initialized the simulation with `B1` and `OP1` .

In iteration:

0. B1 removed, B2 added, OP1 removed
1. OP2 added

2. OP2 removed
