# Supplementary material for: BioDynaMo: a modular platform for high-performance agent-based simulation
Source: Bioinformatics. 2021 Sep 16;38(2):453–60. doi: 10.1093/bioinformatics/btab649 (PMC8723141; doi:10.1093/bioinformatics/btab649)
Supplement: btab649_Supplementary_Data [file btab649_supplementary_data.zip › ST14-randomize-iteration-order.pdf]

Randomize iteration order

Author: Lukas Breitwieser

In this tutorial we show how to randomize the order that BioDynaMo uses in each iteration to process the agents.

Let's start by setting up BioDynaMo notebooks.

In [1]:

```
%jsroot on
gROOT->LoadMacro("${BDMSYS}/etc/rootlogon.C");
```

```
INFO: Created simulation object 'simulation' with UniqueName='simulati
on'.
```

Let's create two helper functions:

- `AddAgents` to add four agents to the simulation
- `print_uid` which prints the uid of the given agent

In [2]:

```
void AddAgents(ResourceManager* rm) {
    for (int i = 0; i < 4; ++i) {
        rm->AddAgent(new SphericalAgent());
    }
}
auto print_uid = [](Agent* a) {
  std::cout << a->GetUid() << std::endl;
};
```

We define an experiment which

1. takes a simulation object as input
2. adds four agents
3. calls `print_uid` for each agent
4. print a seperator so we can distinguish the output of the two different time steps
5. advances to the next time step
6. calls `print_uid` for each agent again

In [3]:

```
void Experiment(Simulation* sim) {
    auto* rm = sim->GetResourceManager();
    AddAgents(rm);

    rm->ForEachAgent(print_uid);
    rm->EndOfIteration();
    std::cout << "-----------------" << std::endl;
    rm->ForEachAgent(print_uid);
}
```

The default behavior of BioDynaMo is to iterate over the agents in the order they were added (not taking multi-threading and load balancing into account). Therefore, we expect to see the same order twice.

In [4]:

```
Experiment(&simulation)
```

```
0-0
1-0
2-0
3-0
-----------------
0-0
1-0
2-0
3-0
```

BioDynaMo also provides a wrapper called `RandomizedRm` , which, as the name suggests, randomizes the iteration order after each iteration. It just takes two lines to add this functionality to the simulation.

In [5]:

```
Simulation simulation("my-sim");
auto* rand_rm = new RandomizedRm<ResourceManager>();
simulation.SetResourceManager(rand_rm);
```

Let's run our experiment again. This time with the simulation which has a randomized resource manager. We expect two different orders.

In [6]:

```
Experiment(&simulation)
```

```
0-0
1-0
2-0
3-0
-----------------
2-0
3-0
0-0
1-0
```
