# Supplementary material for: BioDynaMo: a modular platform for high-performance agent-based simulation
Source: Bioinformatics. 2021 Sep 16;38(2):453–60. doi: 10.1093/bioinformatics/btab649 (PMC8723141; doi:10.1093/bioinformatics/btab649)

Replace mechanical interaction force

Author: Lukas Breitwieser

This tutorial demonstrates how to replace BioDynaMo's default interaction force with a user-defined one. The interaction force is used to calculate forces between agent pairs that are in physical contact with each other.

Let's start by setting up BioDynaMo notebooks.

In [1]:

```
%jsroot on
gROOT->LoadMacro("${BDMSYS}/etc/rootlogon.C");
```

```
INFO: Created simulation object 'simulation' with UniqueName='simulati
on'.
```

In [2]:

```
#include "core/operation/mechanical_forces_op.h"
```

We modify the `simulation_max_displacement` parameter to better visualize the difference of the user-defined force that we will add.

In [3]:

```
auto set_param = [](Param* p) {
    p->simulation_max_displacement = 50;
};
Simulation simulation("my-simulation", set_param);
```

In our experiment we create two overlapping cells and visualize the starting condition.

In [4]:

```
void Experiment() {
    simulation.GetResourceManager()->ClearAgents();
    auto* ctxt = simulation.GetExecutionContext();
    auto* scheduler = simulation.GetScheduler();

    auto* cell1 = new Cell({0, 0, 0});
    auto* cell2 = new Cell({10, 0, 0});
    cell1->SetDiameter(20);
    cell2->SetDiameter(20);
    cell1->SetMass(0.1);
    cell2->SetMass(0.1);

    ctxt->AddAgent(cell1);
    ctxt->AddAgent(cell2);

    scheduler->FinalizeInitialization();
    VisualizeInNotebook();
}
```

Let's run our experiment and have a look at the visualization.

In [5]:

```
Experiment();
```

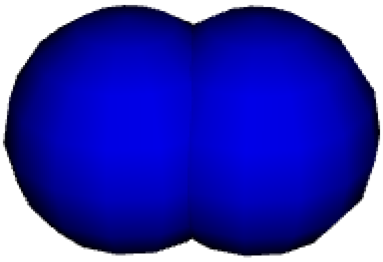

We continue by simulating 10 iterations and observe how the mechanical force pushed the two cells away from each other, until they don't overlap anymore.

In [6]:

```
auto* scheduler = simulation.GetScheduler();
scheduler->Simulate(10);
VisualizeInNotebook();
```

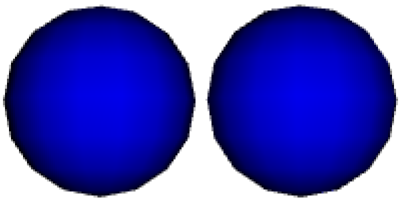

Now we want to add our user-defined force implementation. First, we have to subclass `InteractionForce` and implement our force. In this case, it is an extremely simple (and unrealistic) implementation.

In [7]:

```
class MyInteractionForce : public InteractionForce {
  public:
    MyInteractionForce() {}
    virtual ~MyInteractionForce() {}

    Double4 Calculate(const Agent* lhs, const Agent* rhs) const override {
        if (lhs < rhs) {
            return {100, 0, 0, 0};
        } else {
            return {-100, 0, 0, 0};
        }
    }

    InteractionForce* NewCopy() const override { return new MyInteractionForce(); }
};
```

With the following three lines we instruct BioDynaMo to use our new `MyInteractionForce` instead of the default implementation.

In [8]:

```
auto* myforce = new MyInteractionForce();
auto* op = scheduler->GetOps("mechanical forces")[0];
op->GetImplementation<MechanicalForcesOp>()->SetInteractionForce(myforce);
```

We create the same starting condition as before.

In [9]:

```
Experiment();
```

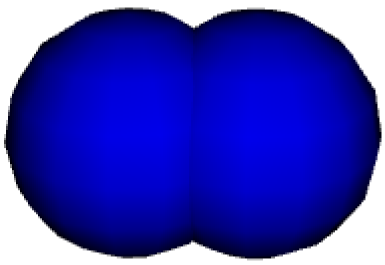

Because `myforce` is so strong, it is sufficient to simulate only one iteration to clearly see its impact.

In [10]:

```
auto* scheduler = simulation.GetScheduler();
scheduler->Simulate(1);
VisualizeInNotebook();
```

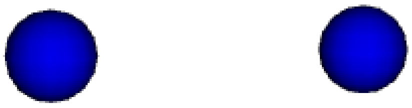

Supplement: btab649_Supplementary_Data [file btab649_supplementary_data.zip › ST15-replace-interaction-force.pdf]
